# Box C/D SNORD116-17 promotes prostate cancer progression by targeting the miR-206/E2F5 axis

**DOI:** 10.1016/j.isci.2026.117806

**Published:** 2026-10-08

**Authors:** Xiuyi Pan, Yifan Kang, Jinjing Zhong, Ting Yu, Linmao Zheng, Yuyan Wei, Junya Tan, Ling Nie, Mengni Zhang, Ni Chen, Qiao Zhou, Xueqin Chen

**Affiliations:** 1 Department of Pathology and State Key Laboratory of Biotherapy, West China Hospital, West China Medical School, Sichuan University, Chengdu, China

**Keywords:** prostate cancer, SNORD116-17, miR-206, E2F5

## Abstract

Small nucleolar RNAs (snoRNAs) are single-stranded non-coding RNAs found in eukaryotic nucleolus, and their aberrant expression is closely related to tumorigenesis. Box C/D SNORD116-17, a member of SNORD116 cluster, is highly expressed in prostate cancer tissues, though its biological function in this malignancy remains uncharacterized. Here, we found that SNORD116-17 is evident, stable, and highly expressed in prostate cancer cells and that its overexpression significantly promoted the proliferation, migration, and invasion of cancer cells. Bioinformatics prediction, RIP, RNA dot blot, and RNA-protein pulldown experiments revealed that miR-206 interacted with SNORD116-17, forming a complex with Argonaute 2. Subsequently, PCR and western blot experiments revealed that both miR-206 and SNORD116-17 regulated the expression of E2F transcription factor 5 (E2F5), a commonly studied oncogene in prostate cancer. In summary, SNORD116-17 contributes to prostate cancer progression by sponging with miR-206 through the ceRNA mechanism and upregulating the expression of E2F5.

## Highlights

• SNORD116-17 is abnormally highly expressed in prostate cancer cells
• SNORD116-17 could competitively bind to miR-206 and Argonaute 2 to form an RNA-protein complex
• SNORD116-17 could obviously promote the prostate cancer cells malignant biological behaviors

## Graphical abstract

**Figure:**
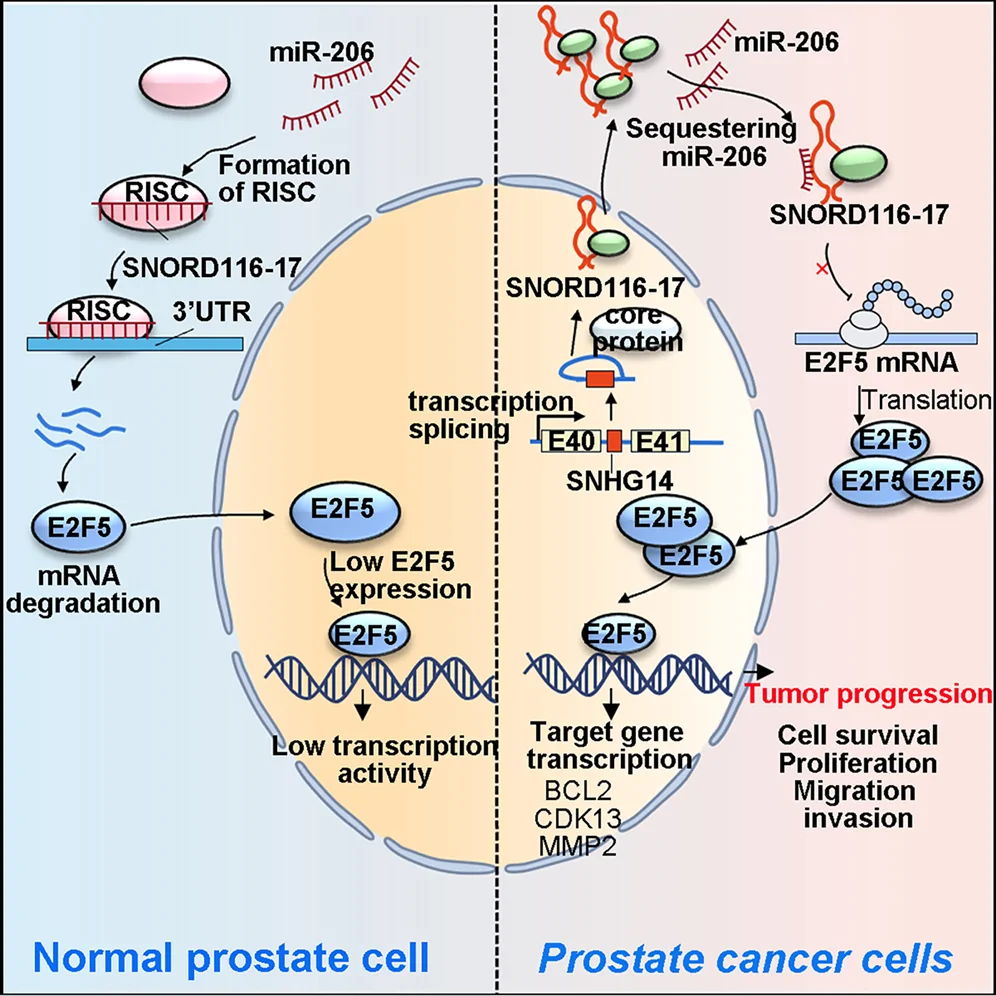

## Teaser

biological sciences; biochemistry; molecular biology; cancer

## Introduction

Prostate cancer (PCa) is the second most common cancer and has the fifth highest mortality rate in males worldwide.^1^ Despite significant advancements in the diagnosis and treatment of PCa, castration-resistant prostate cancer (CRPC) remains a major challenge. In recent years, several aberrantly expressed small nucleolar RNAs (snoRNAs) have been identified in prostate cancer. For example, highly expressed SNORA55 can facilitate PCa progression and is associated with the expression of tumor necrosis factor (TNF) and growth hormone releasing hormone receptor (GHRHR),^2^ and overexpressed SNORA42 can enhance PCa cell proliferation, migration, and epithelial-mesenchymal transition (EMT) and it also correlated with worse survival in metastatic PCa tissues.^3^ However, the underlying molecular mechanism is poorly understood. Therefore, it is essential to explore new mechanisms of snoRNAs in PCa development and progression and to search for new molecular biomarkers and therapeutic targets.

SnoRNAs, approximately 60∼300 nucleotides (nts) in length, are abundant and conserved non-coding RNAs (ncRNAs) in the nucleus.^4^ Most snoRNAs are encoded by the introns of protein-coding genes and non-coding genes called small nucleolar RNA host genes (SNHGs).^5^ Based on their conserved structure, snoRNAs are divided into two major groups: box C/D snoRNAs and box H/ACA snoRNAs. Box C/D snoRNAs contain conserved C box (5′-RUGAUGA-3′) or D box (5′-CUGA-3′) sequences. They typically form small nucleolar ribonucleoprotein particles (snoRNPs) by binding with four core proteins (Nop1p, Nop56p, Nop58p, and Snu13p), and they are primarily responsible for guiding the 2′-*O*-methylation modification of ribosomal RNAs (rRNAs). Box H/ACA snoRNAs contain H box (5′-ANANNA-3′) and ACA box (5′-ACA-3′) sequences. They bind with proteins such as Cbf5p, Gar1p, Nhp2p, and Nop10p, and they are mainly responsible for guiding the pseudouridylation modification of rRNAs.^6^ In addition, snoRNAs can also guide the modification of transfer RNAs (tRNAs),^7^ small nuclear RNAs (snRNAs), and mRNAs (messenger RNAs).^6^

In addition to the classical modification function of snoRNAs, some snoRNAs lack modification targets and are referred to as orphan snoRNAs.^8^ The existence of orphan snoRNAs also suggests that snoRNAs may play more roles. Many studies have found snoRNAs exert unconventional cellular functions, such as performing as a molecule anchor to recruit, bind or relocate proteins and thus play different regulatory roles,^9^^,^^10^^,^^11^^,^^12^ regulating alternative splicing through binding complementary sequences to splicing sites,^13^^,^^14^^,^^15^^,^^16^ being cleaved into sno-derived RNAs (sdRNAs) and participating in the assembly of the RNA-induced silencing complex (RISC) to regulate gene expression.^17^^,^^18^^,^^19^ Though the snoRNA mainly distributed in nucleus, there are now a few reports about snoRNAs regulating gene expression by sponging microRNAs (miRNA) through competitive endogenous RNA (ceRNA) regulatory mechanism in cytoplasm.^20^^,^^21^ The ceRNA regulatory mechanism indicates the RNA with miRNA target sites could co competitively bind to miRNA, inhibiting its negative regulatory effect on mRNA.^22^

Based on a high-throughput small non-coding RNA sequencing result,^23^ we identified that box C/D SNORD116-17, which is a length of 92 nt, is aberrantly overexpressed in PCa cells. The search of the snoRNA relative database revealed that SNORD116-17 belongs to the “orphan RNA” that it has no modification target RNA. In this study, we revealed that SNORD116-17 functions as a molecular sponge, promotes E2F transcription factor 5 (E2F5) expressions by suppressing miR-206, and enhances the malignancy of PCa.

## Results

### SNORD116-17 is highly expressed in PCa cells and tissues

According to a published small non-coding RNA sequencing result,^23^ we have analyzed that the SNORD116 cluster (SNORD116@) is commonly overexpressed in PCa tissues (Figure 1A). We designed the 3′ terminal bases of primers detecting the snoRNAs on the different sites of each snoRNA according to the principle of primers with mismatched 3′ ends efficiently reduce amplification efficiency.^24^ The primers used are listed in Table 1. Those snoRNAs with relatively high copy number and significant difference in PCa tissues and in benign prostate hyperplasia and different groups with different scores were chosen to detect. Based on the principle of aforementioned primer design, the SNORD116-3 and SNORD116-5 cannot be distinguished. But except for SNORD116-17, the others were rarely detected in the PCa cell lines PC-3 and DU145 (Figure 1B).

**Figure 1:**
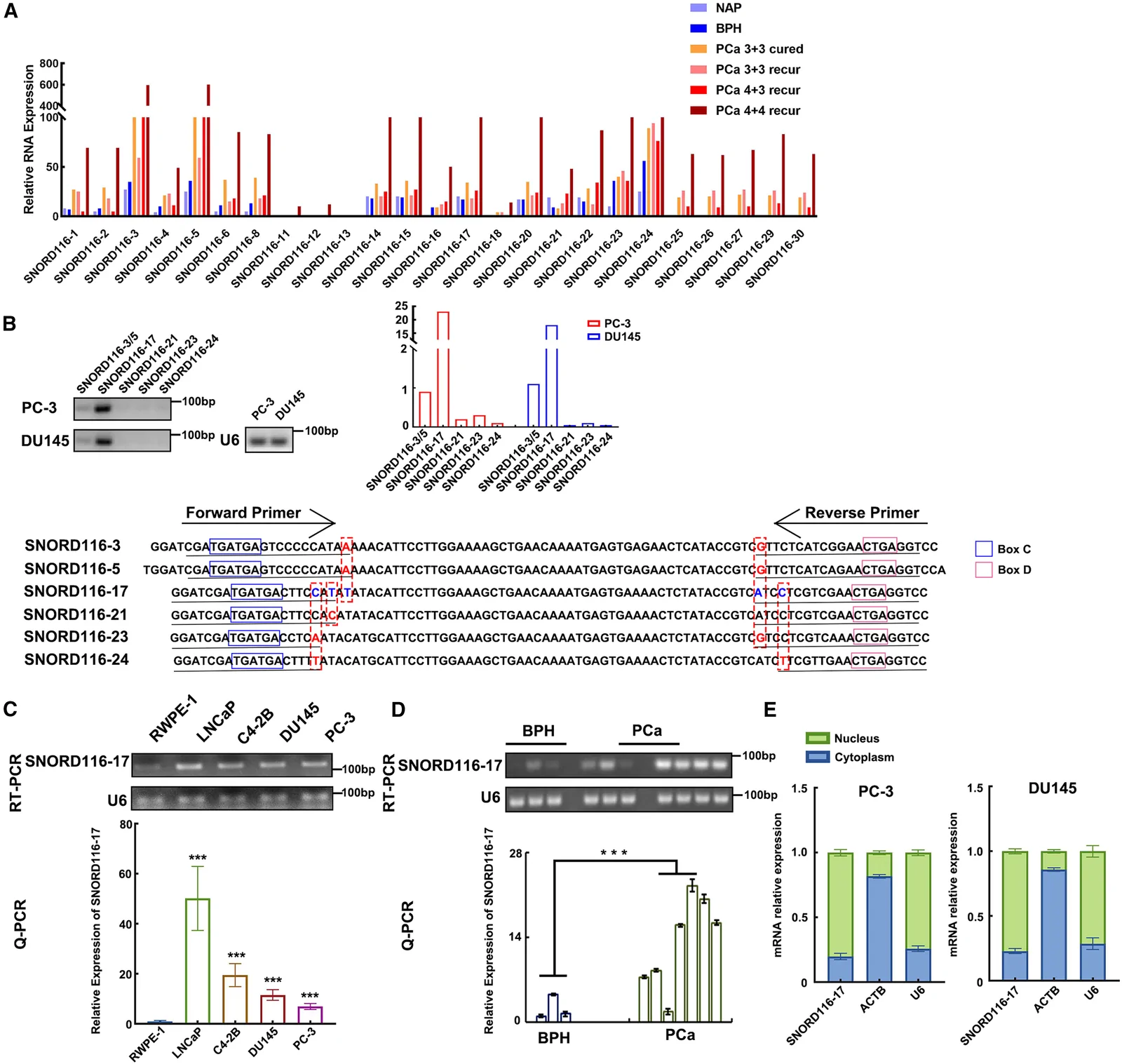
SNORD116-17 is highly expressed in prostate cancer cells and tissues (A) According to a published small non-coding RNA sequencing result, the SNORD116 cluster, which are detected in BPH and PCa tissues are presented in the figure. NAP, normal adult prostate; BPH, benign prostate hyperplasia; PCa, prostate cancer. (B) Top: SNORD116-3/5, SNORD116-17, SNORD116-21, SNORD116-23, and SNORD116-24 were amplified by RT-PCR. The lines represent the primers’ sequences. PCR was performed with 1 μL cDNA reverse-transcribed from 2 μg RNA. After the cycles of 30, SNORD116-17 was significantly detected, but others only weakly appeared. U6 band was detected after 28 cycles. Bottom: different 3′ ends of each primer were shown in red, compared to the corresponding bases of SNORD116-17 shown in blue. (C) RNA levels of SNORD116-17 in prostate cancer cells (LNCaP, C4-2B, DU145, PC-3) were much higher compared to RWPE-1 by reverse transcription PCR (RT-PCR) **(**top**)** and reverse transcription real-time quantitative PCR (qPCR) (bottom). Fold changes are normalized to U6 snRNA levels. (D) RNA levels of SNORD116-17 in PCa tissues were much higher than that in BPH detected by RT-PCR (top) and qPCR (bottom). PCa, prostate cancer; BPH, benign prostate hyperplasia. (E) Nuclear and cytoplasmic RNAs extracted from PC-3 and DU145 cells were assayed by RT-PCR and qPCR. The cytoplasm-distributed ACTB and the nuclear-distributed U6 were used as markers. The ratios of RNA relative expression level of SNORD116-17, Actin and U6 account for 80%, 18%, and 74% in nucleus of PC-3, 77%, 14%, and 71% in nucleus of DU145, respectively. Unless indicated, data are represented as mean ± SEM. ∗*p* < 0.05; ∗∗*p* < 0.01; ∗∗∗*p* < 0.001, unpaired two-tailed Student’s *t* test.

**Table 1:** All primers used in this study

Name	Sequences
SNORD116-17	5′- TCTAGAGGATCGATGATGACTTCCAT -3′
–	5′- TCTAGAGGACCTCAGTTCGACGAGGAT -3′
SNORD116-3/5	5′-GATGAGTCCCCCATAA-3′
–	5′-CCTCAGTTCCGATGAGAAC-3′
SNORD116-21	5′-GGATCGATGATGACTTCCAC-3′
–	5′-CAGTTCGACGAGGAT-3′
SNORD116-23	5′-TCGATGATGACCTCA-3′
–	5′-CAGTTTGACGAGGAC-3′
SNORD116-24	5′-TCGATGATGACTTTT-3′
–	5′-CCTCAGTTCAACGAA-3′
SNORD116-17-pWT	5′-GGGGTACCCCTGTGCGATCCTTGGAGATGC-3′
–	5′-CCCTCGAGGGAACCAAGCTGTCTCTCAGCC-3′
SNORD116-17-T7WT	5′-GGGGTACCCCGGATCGATGATGACTTCCATA-3′
–	5′-CCCTCGAGGGGGACCTCAGTTCGACGAGGAT-3′
SNORD116-17-T7mut	5′-GGGGTACCCCGGATCGATGATGACTTCCATATAATGTAAGGTT-3′
–	5′-CCCTCGAGGGGGACCTCAGTTCGACGAGGAT-3′
MiR-206-FP	5′-CGGCGTGGAATGTAAGGA-3′
MiR-1-3p-FP	5′-CGGCGTGGAATGTAAAGA-3′
MiR-RP	5′-GTGCAGGGTCCGAGGT-3′
MiR-1-3p-RT	5′-GTCGTATCCAGTGCAGGGTCCGAGGTATTC GCACTGGATACGACATACATA-3′
MiR-206-RT	5′-GTCGTATCCAGTGCAGGGTCCGAGGTATTCG CACTGGATACGACCCACACA-3′
E2F5	5′-CACCTTCTGGTACACAACTGG-3′
–	5′-GGGCTTAGATGAACTCGACTC-3′
CORO1C	5′-ATGCGTTGGAAGGACACGAT-3′
–	5′-CAGTCTTGTGCAGAGGGAGG-3′
CXCL11	5′-ATGAGTGTGAAGGGCATGGC-3′
–	5′-TCACTGCTTTTACCCCAGGG-3′
CDK4	5′-GCCCTCAAGAGTGTGAGAGTC-3′
–	5′-CACGAACTGTGCTGATGGGA-3′
U6	5′- CTCGCTTCGGCAGCACA-3′
–	5′- TGGAACGCTTCACGAATTTG-3′
ACTB	5′-CTGGCACCACACCTTCTACAATG-3′
–	5′-CCTCGTAGATGGGCACAGTGTG-3′
SNORD116-17-mut	5′-TGATGACTTCCATATAATGTAAGGTTGG AAAGCTG-3′
–	5′-CAGCTTTCCAACCTTACATTATATGGAA GTCATCA-3′
E2F5-3′UTR-WT	5′-GCTCTAGAGCAGTGCCTTCTGTTTTAG CACT-3′
–	5′-GCTCTAGAGCTGTACAGGCATTGGC ACATT-3′
E2F5-3′UTR-mut	5′-TGACTTCTGTGTAAGGTCTTTCCT-3′
–	5′-AGGAAAGACCTTACACAGAAGTCA-3′

Reverse-transcription PCR (RT-PCR) and qPCR demonstrated that SNORD116-17 was highly expressed in the PCa cell lines. Compared with the normal prostate epithelial cell line RWPE-1, the relative expression levels of SNORD116-17 in LNCaP, C4-2B, DU145, and PC-3 cells were 52.5, 27.4, 12.1, and 7.3 times, respectively (Figure 1C). Reverse-transcription PCR and qPCR also revealed significantly higher expression levels of SNORD116-17 in prostate cancer (PCa) tissues than in benign prostatic hyperplasia (BPH) tissues (Figure 1D). The average expression level of SNORD116-17 in PCa is about 5.79 times that in BPH. Cell fractionation experiments showed that SNORD116-17 RNA was can be detected in both the nucleus and cytoplasm of PCa cells, although it is mainly located in nucleus (Figure 1E).

### Upregulated SNORD116-17 promoted PCa cell proliferation, migration, and invasion

To ascertain the effect of SNORD116-17 on the malignant properties of PCa cells, we performed several *in vitro* assays. First, we examined the effects of intervening (knockdown by ASO-SNORD116-17 or artificial overexpression by SNORD116-17 plasmid) with SNORD116-17 in cells. qPCR results showed ASO-SNORD116-17-02 has the strongest effect, knocking down SNORD116-17 RNA by more than 70% in PC-3 and DU145 cells. The pcDNA3.1 plasmids, which are the overexpressed precursor of SNORD116-17, also can effectively increase the levels of SNORD116-17 RNA by more than 20 times (Figure 2A). Thus, we choose the ASO-02 and pcDNA3.1 plasmid to conduct the subsequent experiments. PC-3 are the cells of PCa that metastasized to bone, while DU145 is the cells of PCa metastasized to brain. They are the androgen-independent tumor cells. Then the overexpression of SNORD116-17 in PC-3 and DU145 cells significantly promoted cell metabolism and DNA synthesis, thus promoted the cell proliferation, as determined by a CCK-8 assay (Figure 2B) and an 5-ethynyl-2′-deoxyuridine (EdU) assay (Figure 2C). Knockdown of SNORD116-17 significantly attenuates cell metabolism and DNA synthesis in PC-3 and DU145 cells, thus inhibited the cell proliferation. In addition, wound healing, transwell migration, and invasion assays are widely used to evaluate the migration and invasion signatures of *in vitro*-cultured cells. SNORD116-17 upregulation markedly enhanced the migratory and invasive capacities of PC-3 and DU145 cells, as determined by wound healing assays (Figure 2D) and transwell migration and invasion assays (Figure 2E). In contrast, we knocked down SNORD116-17 in PC-3 and DU145 cells, which strongly inhibited cell migration, and invasion (Figures 2C–2E).

**Figure 2:**
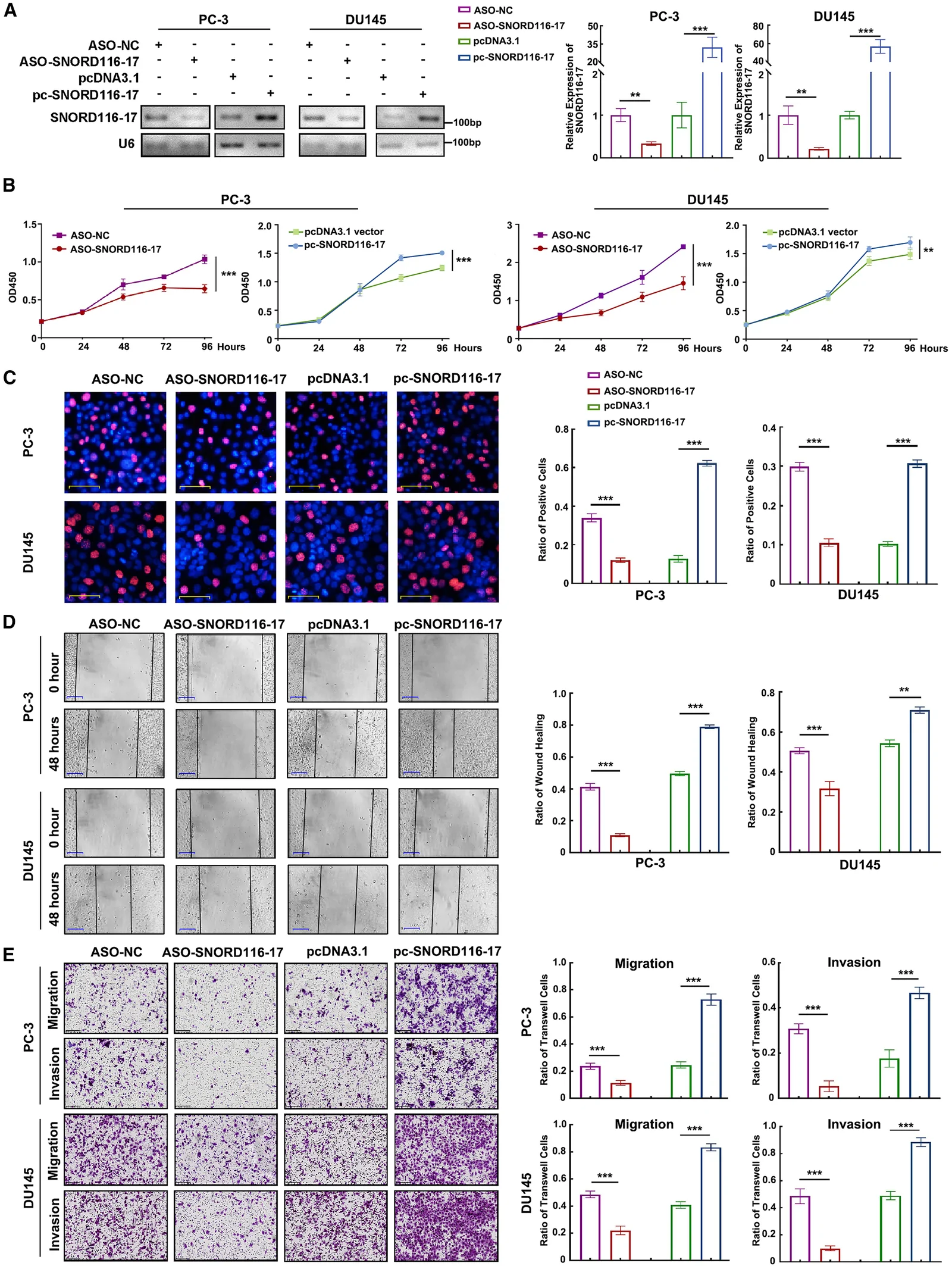
Upregulated SNORD116-17 promoted PCa cell proliferation, migration, and invasion (A) The intervention (knockdown or overexpression) effects of SNORD116-17 in PC-3 and DU145 cells. (B) CCK-8 assays showed upregulation of SNORD116-17 promoted the proliferative capacities of PC-3 and DU145 cells and downregulation of SNORD116-17 attenuated the proliferative capacities of PC-3 and DU145 cells. (C) EdU assays showed upregulation of SNORD116-17 promoted the DNA synthesis (associated with cell proliferation) of PC-3 and DU145 cells and downregulation of SNORD116-17 inhibited DNA synthesis of PC-3 and DU145 cells. of PC-3 and DU145 cells. Scale bars, 100 μm. (D) Schematic representation showed upregulation of SNORD116-17 promoted the migration capacities of PC-3 and DU145 cells and downregulation of SNORD116-17 attenuated the migration capacities of PC-3 and DU145 cells by using wound healing assays. Scale bars, 200 μm. (E) Transwell migration and invasion assays showed upregulation of SNORD116-17 promoted the migration and invasion capacities of PC-3 and DU145 cells and downregulation of SNORD116-17 attenuated the migration and invasion capacities of PC-3 and DU145 cells. Scale bars, 200 μm. Unless indicated, data are represented as mean ± SEM. ∗*p* < 0.05; ∗∗*p* < 0.01; ∗∗∗*p* < 0.001, unpaired two-tailed Student’s *t* test.

### Bioinformatic analysis and experimental validation of potential interactions between SNORD116-17 and miR-206

Next, we conducted qPCR and the results revealed that intervening with SNORD116-17 has no noticeable influence on the expression of its four core binding proteins (Fibrillarin/Nop1p, NOP56, NOP58, and Snu13p/p15.5kd)^25^ or its host gene SNHG14 (Figure 3A), suggesting that it may play other roles. We conducted bioinformatics analysis via the starBase Database: https://rnasysu.com/encori/, and the results indicated that SNORD116-17 has a latent binding site for miR-206 and miR-1-3p (Figure 3B). Specifically, we found that the complementary sequence (UACAUUCC) to miR-206 seed sequence (from 2 to 9 bases) is situated within the 24–31 base region of SNORD116-17. Moreover, this particular complementary sequence is precisely located between the box C and box D motifs of SNORD116-17. We also conducted a bioinformatics analysis of the SNORD116 family to predict RNAs that have potential complementary interactions with miR-206. The results revealed that 12, 14, 15, 18, 19, 20, and 21 subtypes of SNORD116 family also have the complementary sequences with miR-206. We subsequently conducted qPCR in PC-3, DU145, C4-2B, LNCaP, and normal human prostate epithelial cell line RWPE-1 cell lines and found that the expression of miR-206 can be detected in cells (Figure 3C), but the expression of miR-1-3p was quite low (data not shown).

**Figure 3:**
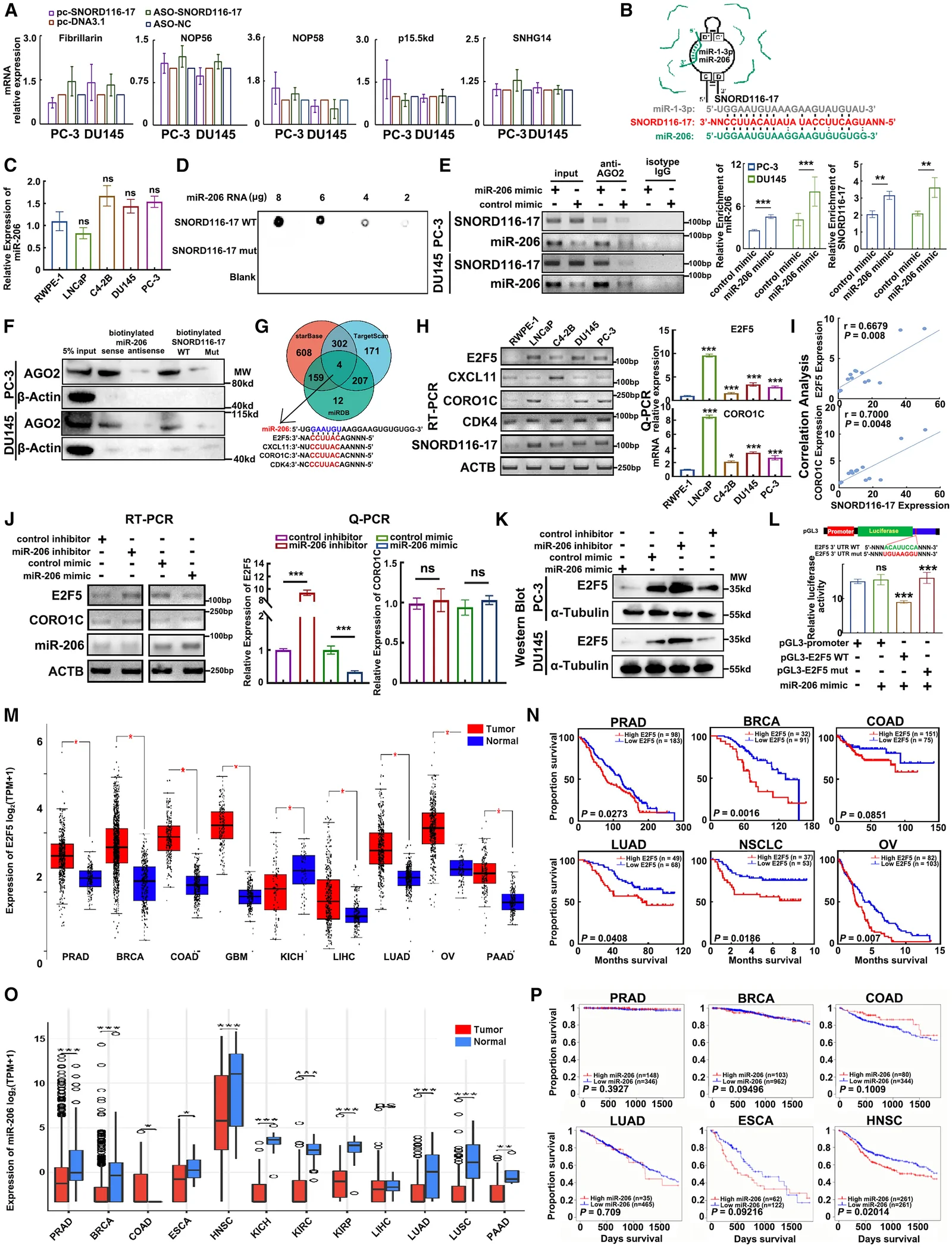
SNORD116-17, miR-206, and AGO2 constitute a complex, and miR-206 could inhibit the expression of E2F5 by targeting its 3′ UTR (A) qPCR revealed that SNORD116-17 has no noticeable influence on the expression of its binding proteins or its host genes. (B) Bio-informatics analysis indicated SNORD116-17 has a potential binding site with miR-1-3p and miR-206. The sequence is located from 24 to 31 bases of SNORD116-17. (C) The expression levels of miR-206 in different prostate cells (RWPE-1, LNCaP, C4-2B, DU145, PC-3) were detected by qPCR. (D) RNA dot blot analysis showed concentration-dependent binding of biotin-labeled miR-206 with wild-type SNORD116-17, but not with mutated SNORD116-17. (E) RIP assays showed SNORD116-17 and miR-206 are in the anti-AGO2 immunoprecipitates from PC-3 and DU145 cells. (F) RNA-protein pulldown assays indicated AGO2 could be pulled down by biotinylated miR-206 and SNORD116-17. (G) Bio-informatics analysis indicated E2F5, CXCL11, CORO1C, and CDK4 contain a complementary sequence (marked by red) of miR-206 seed region (marked by blue). (H) The mRNA levels of E2F5, CORO1C, CDK4, and CXCL11 in different prostate cells (RWPE-1, LNCaP, C4-2B, DU145, PC-3) were detected by RT-PCR (left) and qPCR (right). (I) Spearman’s rank correlation analysis was conducted between the expression of SNORD116-17 and E2F5 or CORO1C. (J) The effect of miR-206 on E2F5 and CORO1C mRNA expression was detected by RT-PCR (left) and qPCR (right). (K) Western blot (WB) was used to detect the effect of miR-206 on the expression of E2F5 in PC-3 and DU145 cells. (L) Dual-luciferase reporter assays showed miR-206 mimic suppressed reporter gene activity of wild-type pGL3-E2F5 3′ UTR construct (shown in diagram previously), but not of mutated pGL3-E2F5 3′ UTR plasmid (shown in diagram above). pGL3-promoter was the blank plasmid control. (two-way ANOVA and Student’s *t* test). (M) Analysis of E2F5 mRNA expression in multiple types of cancer from TCGA dataset. (N) Kaplan-Meier survival analysis of the overall survival (OS) rates of patients with prostate adenocarcinoma (PRAD), breast invasive carcinoma (BRCA), colon adenocarcinoma (COAD), lung adenocarcinoma (LUAD), non-small cell lung cancer (NSCLC), and ovarian serous cystadenocarcinoma (OV) with low and high E2F5 mRNA expression. (O) Analysis of miR-206 expression in multiple types of cancer from TCGA dataset. (P) Kaplan-Meier survival analysis of the OS rates of patients with PRAD, BRCA, COAD, LUAD, esophageal carcinoma (ESCA), and head and neck squamous cell carcinoma (HNSC) with low and high miR-206 expression. Unless indicated, data are represented as mean ± SEM. ns, no significant, ∗*p* < 0.05; ∗∗*p* < 0.01; ∗∗∗*p* < 0.001, unpaired two-tailed Student’s *t* test.

Many previous studies have revealed that the Argonaute 2 (AGO2) protein binds with miRNAs.^26^^,^^27^ To investigate the interactions among SNORD116-17, miR-206, and AGO2, we performed RNA immunoprecipitation (RIP) and RNA dot blot assays, and the results revealed that SNORD116-17 could combine with wild-type miR-206 in a concentration-dependent manner (Figure 3D) and miR-206 and SNORD116-17 were present in the protein-RNA complexes precipitated by the anti-AGO2 antibody (Figure 3E). Additionally, we confirmed that the AGO2 protein could be pulled down by sense miR-206 and wild-type SNORD116-17 probes (Figure 3F). These experiments suggested that SNORD116-17, miR-206, and AGO2 all exist in the same complex.

### miR-206 inhibits the expression of E2F5 by targeting its 3′ UTR

We predicted miR-206 target genes via the starBase Database: https://rnasysu.com/encori/, the TargetScanHuman Database: https://www.targetscan.org/, and the miRDB Database (microRNA database): https://mirdb.org/ and identified the mRNAs of E2F5, CXCL11, CORO1C, and CDK4, which contain the complementary sequence of the miR-206 seed sequences (nts 2–8 of the miRNA) (Figure 3G). We subsequently conducted reverse-transcription PCR and qPCR in PC-3, DU145, C4-2B, LNCaP, and RWPE-1 cells and discovered that expression trends of E2F5 and CORO1C are consistent with those of SNORD116-17 (*p* < 0.05), which are significantly upregulated in PCa cells (Figures 3H and 3I).

To further prove that miR-206 inhibits E2F5 or CORO1C expression, we artificially synthesized the miR-206 inhibitor and mimic. Reverse-transcription PCR and qPCR analyses were performed to examine the expression levels of candidate genes in cancer cells after upregulation or silencing of miR-206 expression (Figure 3J). miR-206 knockdown significantly increased E2F5 expression, and E2F5 expression considerably decreased after overexpressing miR-206. The expression level of CORC1C did not notably change when miR-206 was added. Western blot (WB) assays also confirmed the negative correlation between miR-206 and E2F5 expression (Figure 3K).

miRNAs commonly bind to the 3′ UTR of target mRNAs and then decrease their stability or block translation.^27^^,^^28^ We constructed pGL3-promoter plasmids containing the binding site of miR-206 on the E2F5 3′ UTR and the corresponding mutant sequence for the dual-luciferase reporter assay. The miR-206 mimic reduced the luciferase activity of the pGL3-E2F5-3′ UTR-WT plasmid but did not influence the activity of the pGL3-E2F5-3′ UTR-mut plasmid (Figure 3L), suggesting that miR-206 interacts with E2F5 at the binding site in the 3′ UTR and might influence the stability of the mRNA.

Then, we analyzed relative E2F5 transcription in pan-cancer tissue from the The Cancer Genome Atlas (TCGA) dataset analysis website UALCAN (The University of Alabama at Birmingham Cancer data analysis Portal): https://ualcan.path.uab.edu/analysis.html, and results showed that E2F5 mRNA was significantly higher in the majority of cancers (Figure 3M). In addition, E2F5 expression exhibited relatively worse survival than patients with lower E2F5 levels in some types of tumors, such as prostate adenocarcinoma (PRAD) (Figure 3N).

In correspondence, the data from the CancerMIRNome Database: https://bioinfo.jialab-ucr.org/CancerMIRNome/ shows miR-206 was significantly lower in the majority of cancers (Figure 3O), and miR-206 expression exhibited relatively better survival than patients with lower miR-206 levels in several types of tumors but not in PRAD (Figure 3P).

### SNORD116-17 functions as a ceRNA through sponging miR-206 and then increases the expression level of E2F5

We have predicted that SNORD116-17 has a complementary sequence of miR-206, suggesting SNORD116-17 might antagonize the function of miR-206. When SNORD116-17 expression was artificially upregulated, the reverse-transcription PCR and qPCR results revealed a significant increase in the level of E2F5 expression, and E2F5 expression level was significantly decreased after SNORD116-17 expression was knocked down (Figure 4A). Rescue PCR assays also confirmed SNORD116-17 upregulates E2F5 expression and miR-206 blocks the increase (Figure 4B), as did WB assays (Figure 4C).

**Figure 4:**
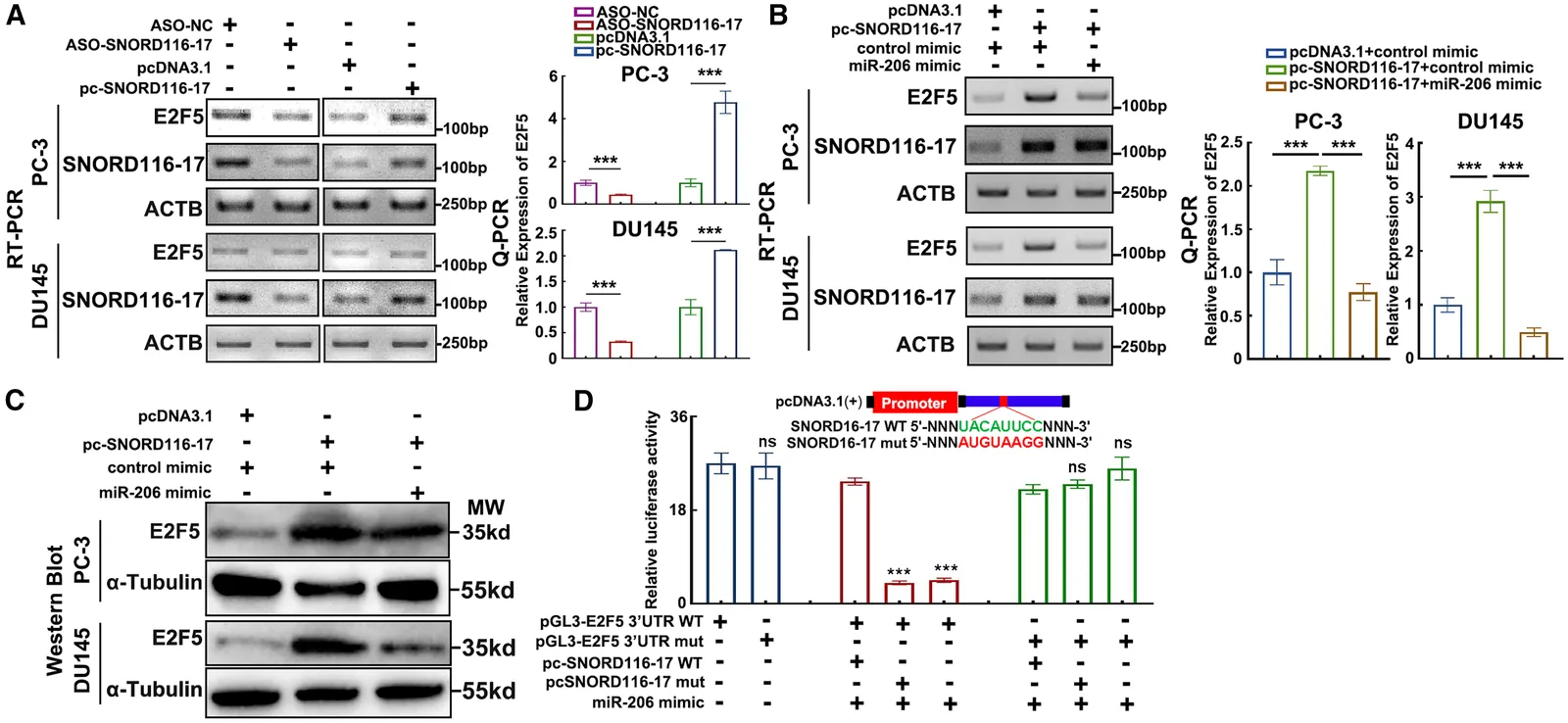
SNORD116-17 functions as ceRNA through sponging miR-206 then increases expression level of E2F5 (A) The effect of SNORD116-17 on E2F5 mRNA expression, detected by RT-PCR (left) and qPCR (right). (B) Rescue assays of overexpressing SNORD116-17 and miR-206, the effect of E2F5 expression was detected by RT-PCR (left) and qPCR (right). (C) Rescue assays of overexpressing SNORD116-17 and miR-206, the effect of E2F5 expression was detected by WB. (D) The dual-luciferase reporter assays showed miR-206 mimic suppressed reporter gene activity of wild-type pGL3-E2F5 3′ UTR construct, but not of mutated pGL3-E2F5 3′ UTR plasmid. Along with wild-type pcDNA3.1-SNORD116-17 (shown in the diagram), not mutated pcDNA3.1-SNORD116-17 (shown in the diagram), enhanced reporter gene activity of wild-type pGL3-E2F5 3′ UTR plasmid, but not of mutated pGL3-E2F5 3′ UTR construct. (two-way ANOVA and Student’s *t* test). Unless indicated, data are represented as mean ± SEM. ns, no significant; ∗*p* < 0.05; ∗∗*p* < 0.01; ∗∗∗*p* < 0.001, unpaired two-tailed Student’s *t* test.

Subsequently, we artificially over-expressed wild-type and mutant SNORD116-17 RNA and found that treatments with miR-206 mimics and SNORD116-17-WT plasmid, not SNORD116-17-mut plasmid, resulted in a significant increase in the luciferase activity of pGL3-E2F5-3′ UTR-WT plasmid but not of the pGL3-E2F5-3′ UTR-mut plasmid (Figure 4D). The primers used for plasmid construction are summarized in Table 1. The previous results indicate that SNORD116-17 attenuates the inhibitory role of miR-206 through binding to it, and eventually upregulates the expression of E2F5.

### Overexpression of SNORD116-17 promotes tumor growth

The stable overexpression of SNORD116-17 (SNORD116-17-OE) and the control DU145 cells were injected subcutaneously (s.c.) into nude mice, and tumors and mice were observed regularly. After 40 days, the nude mice were euthanized and the tumors were isolated. All the tumors grew in the SNORD116-17-OE xenograft group, and 5 tumors grew in the control xenograft group (Figure 5A). The results showed that the tumors in the SNORD116-17-OE xenograft group grew faster than those of the control group. qPCR results showed the expression levels of SNORD116-17 and E2F5 obviously higher than that of control group (Figures 5B and 5C). And Ki-67 staining detection of the xenografts demonstrated overexpression of SNORD116-17 can remarkably promote cell proliferation (Figure 5D).

**Figure 5:**
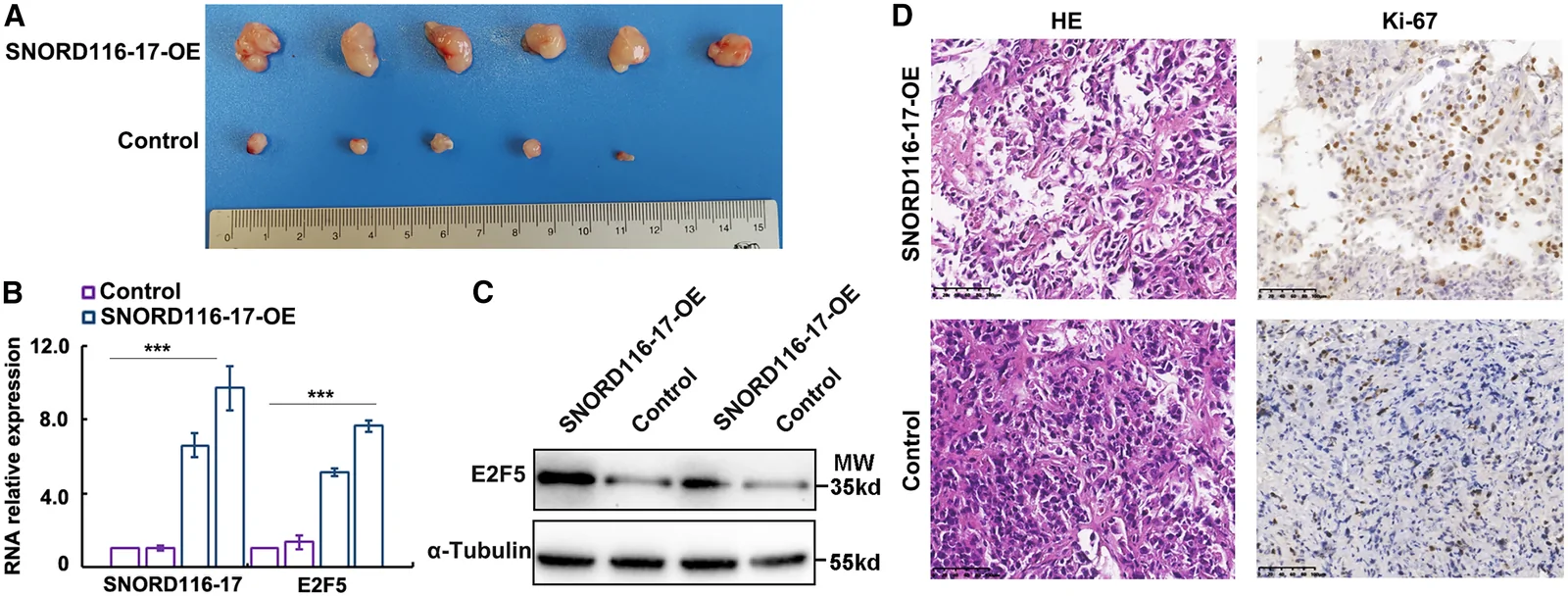
Overexpression of SNORD116-17 promotes tumor growth (A) The average tumor sizes are obviously increased by overexpression of SNORD116-17 in xenograft models. Average tumor volume of SNORD116-17-OE xenograft group: 840 ± 80.39 mm^3^, average tumor volume of control xenograft group: 81 ± 7.7 mm^3^. (B) qPCR results show the SNORD116-17 expression level in SNORD116-17-OE xenograft group is about 8.16 times than that of control group and E2F5 mRNA expression level in SNORD116-17-OE xenograft group is about 6.4 times than that of control group (*n* = 6, *n* = 5). Data are represented as mean ± SEM. ∗∗∗*p* < 0.001, unpaired two-tailed Student’s *t* test. (C) WB results show the E2F5 protein level in SNORD116-17-OE xenograft group is much higher than that of control group. (D) HE and IHC staining analysis of xenograft tumor tissue shows overexpression of SNORD116-17 promote tumor cell proliferation. The Ki-67 index of SNORD116-17-OE xenograft group is about 30%, and Ki-67 index of control group is about 10%.

## Discussion

The SNORD116@ belongs to the box C/D snoRNAs.^24^ SNORD116 lacks known modification targets so far and is referred to as an orphan snoRNA. It has been proven to be associated with Prader-Willi syndrome (PWS),^25^^,^^26^ which is a rare and complex genetic disorder that affects multiple systems in the body, including metabolic, endocrine, and neurologic functions, and it is characterized by severe hypotonia and feeding difficulties in infancy, which lead to developmental delays. It has been well established that SNORD116-17 is associated with the pathogenesis of PWS,^29^^,^^30^ but knocking out the SNORD116 gene cluster in mouse models cannot fully recapitulate the human PWS phenotype.

It has also been reported that the loss of SNORD116 enhances some aspects of the developmental timing of neuronal cells and alters mRNA expression like MAGEL2.^31^ In osteoarthritis chondrocytes, the expression of SNORD116 is higher than normal cells, which may be associated with osteoarthritis.^32^ However, research on SNORD116 in tumors is relatively scarce, it has found that SNORD116-26 is under-expressed in lung tumor-initiating cells by high-throughput sequencing^33^; in non-small cell lung cancer (NSCLC), low expression of SNORD116 in exosomes is associated with poor prognosis^34^; in electroretinography (ERG)-related leukemia, a subtype of B cell precursor acute lymphoblastic leukemia (BCP ALL), the expression of SNORD116 is upregulated^35^; in gastric cancer, low expression of SNORD116-4 is associated with poor prognosis.^36^ The previous results suggest that SNORD116 may play a regulatory role in the occurrence and progression of tumors. To date, in human cancer, there is no literature about SNORD116-17. In this study, we firstly discovered that SNORD116-17 is abnormally highly expressed in PCa cell lines. Further research revealed that SNORD116-17 regulates the expression of the target gene E2F5 and significantly influences the biological functions of PCa cell lines.

In a high-throughput sequencing article on PCa, the SNORD116@ is commonly overexpressed in tumor tissues,^23^ suggesting its potential involvement in the tumorigenesis of PCa. We have detected in PCa tissues and cells and found that the expression of SNORD116-17 is much higher in PCa tissues and cells compared with BPH and RWPE-1 cells, respectively. As with other snoRNAs,^3^^,^^37^ SNORD116-17 also mainly located in the nucleus. However, an increasing number of literatures that indicates that although snoRNA is present in relatively low amounts in the cytoplasm, a certain or even significant role in the cytoplasm have been appreciated. Such as, SNORD50A and SNORD50B can directly bind to K-Ras and suppress the activity in cytoplasm in human cancer.^38^ And in metabolic stress, the snoRNA in the cytoplasm also exert a crucial function.^39^ There are some snoRNAs were demonstrated to affect the content of target molecules or the signal pathways,^40^^,^^41^^,^^42^ but potential mechanisms are still unclear. In the study, we have found intervene of SNORD116-17 can obviously affect the proliferation, migration, and invasion of PCa cells. In xenograft models, overexpression of SNORD116-17 can obviously increase the xenograft tumor Ki-67 index and promote tumor growth. In the suggested snoRNA, despite its low abundance in the cytoplasm, its abnormal expression may exert significant biological functions.

In this research, we have discovered that SNORD116-17 can interact with miR-206. The encoding gene of miR-206 locates in the 6p12.2 region of human chromosome 6. Initially, miR-206 was described as a muscle-specific miRNA.^43^ Later, it was gradually discovered that miR-206 regulates various cellular functions in different tissues by binding to and inhibiting hundreds of target mRNAs. Several studies found that miR-206 is underexpressed in many types of tumors such as breast cancer,^44^ cervical cancer,^45^ lung cancer,^46^ and clear cell renal cancer,^47^ and it functions as a tumor suppressor by inhibiting oncogenes expression or tumor-associated signaling pathways, such as MKL1/IL11, Notch3, and C-Myc. In PCa, miR-206 is also underexpressed, and it is reported that nuclear factor erythroid 2-related factor 2 (NRF2) exerts transcriptional regulatory effects to suppress the expression of miR-206.^48^ The low expression of miR-206 in PCa suggests that it may exert tumor-suppressive effects. For instance, circular RNA (circRNA) circ0057558 upregulates the expression of USP33 by sponging miR-206, which in turn upregulates the expression of c-Myc to promote tumor cell proliferation.^49^ LncRNA AP000842.3 can also upregulate the expression of NFAT5 through a similar mechanism, thereby promoting tumor cell proliferation and reducing sensitivity to cuproptosis.^50^ In addition, miR-206 can also inhibit the EMT and metastatic ability of cells by downregulating the expression of ANXA2.^51^ In summary, miR-206 can be considered a tumor suppressor gene in PCa.

The interaction between SNORD116-17, miR-206, and AGO2 suggests ceRNA regulatory mechanism. Plenty of ncRNAs, mainly long non-coding RNAs (lncRNAs) and circRNAs have been shown to widely regulate gene expression through the ceRNA mechanism.^52^^,^^53^^,^^54^ It involves the interaction between ncRNAs and miRNAs, thereby reducing the binding of miRNAs to target mRNAs and attenuating their inhibitory effects. In recent years, a few studies have reported that snoRNAs can exert a ceRNA role to regulate gene expression, for example, SNORA73 can promote psoriasis progression through sponging miR-3074-5p and upregulating PBX1^21^ and SNORA75 can promote endometrial receptivity by binding miR-146a-3p and upregulating ZNF23.^20^ Our study has found although the expression level of SNORD116-17 in the cytoplasm accounts for 20% of the total cellular content, it still can bind with miR-206 in PCa, thereby leading to the upregulation of the expression of miR-206’s target E2F5.

E2F5 belongs to the E2F family, transcription factors of which are often associated with the regulation of the cell cycle.^55^ E2F5 is overexpressed in various tumors and is associated with poor prognosis, including PCa. Current research findings indicate that the expression of E2F5 is regulated by a variety of factors, primarily under the negative regulation of miRNAs, such as miR-132,^56^ miR-493–3p,^57^ and miR-154–5p.^58^ Additionally, some ncRNAs can regulate the expression of E2F5 through the ceRNA mechanism, such as circCDK13 promotes the expression of E2F5 by binding miR-212-5p and miR-449a,^59^ and LINC01980 promotes the expression of E2F5 by sponging miR-376b-5p.^60^ Furthermore, the transcription factor MYCN can promote the transcription of E2F5,^61^ METTL3 enhances the stability of E2F5 mRNA through m^6^A modification,^62^ and IMP3 binds to E2F5 mRNA to prolong its half-life.^63^ As a transcription factor, E2F5 has been reported to regulate the expression of various genes in different tissues or cells, including in PCa, such as cell cycle-related CDK13,^59^ CDK2, and CDK6,^61^ apoptosis-related BCL2,^64^ cell metastasis-related MMP2, MMP9, and TFPI2,^65^ ubiquitination protein UBE2T,^66^ NF-κB signaling pathway activator NIK,^67^ and pro-oncogenic ncRNA LINC00146.^68^ E2F5 can also regulate the cell cycle by phosphorylating SMAD3.^69^ *In vitro*, our research found that SNORD116-17 upregulates the expression of E2F5 mRNA and protein through the ceRNA mechanism. And *in vivo*, our experiments also further confirmed that the expression of E2F5 mRNA and protein increased accompany by SNORD116-17 was artificially upregulated. It eventually enhanced the proliferation, migration, and invasion and tumor growth capabilities of PCa cells.

In summary, our study demonstrated that the box C/D snoRNA SNORD116-17 is highly expressed in PCa cell lines. Overexpressing SNORD116-17 can facilitate the proliferation, migration, and invasion capabilities of PCa cells. Through further research, we found that SNORD116-17 upregulates E2F5 expression and promotes PCa progression by sponging miR-206 through a ceRNA mechanism, which provides new ideas for discovering new methods for PCa diagnosis and treatment.

### Limitations of the study

This study has several limitations. Firstly, our clinical sample only contained 3 BPH samples and 7 PCa tissues with a limited sample size. Larger clinical cohorts are required to systematically evaluate the expression differences of SNORD116-17 in PCa. Secondly, we characterized the SNORD116-17/miR-206/E2F5 ceRNA axis. Whether SNORD116-17 regulates malignant behaviors of PCa cells through other independent molecular pathways remains unclear. Finally, we only observed stable upregulation of SNORD116-17 rather than other SNORD116 members in PCa cells. The upstream molecular drivers selectively inducing SNORD116-17 overexpression remain unclear, which needs further in-depth investigation.

## Resource availability

### Lead contact

Requests for further information, resources and reagents should be directed to and will be fulfilled by the lead contact, Xueqin Chen (chenxueqin1980@wchscu.cn).

### Materials availability

This study did not generate new datasets.

### Data and code availability

All data reported in this paper will be shared by the lead contact upon request. This article does not report original code. Any additional information required to reanalyze the data reported in this article is available from the lead contact upon request.

## Acknowledgments

This work was supported by the 10.13039/501100001809National Natural Science Foundation of China (grant numbers 82273047, 82273073, 82203280, 82203315, and 82472650) and the Sichuan Province Science and Technology support program (2025ZNSFSC1889, 2025ZNSFSC0552, and 2025ZNSFSC0786).

## Author contributions

X.P. and Y.K. designed the experiments, constructed the plasmids, analyzed the data, and wrote the manuscript; X.P. and J.T. performed the experiments; L.Z., and J.Z. conducted the bioinformatic analysis and statistical analysis; T.Y. and Y.W. provided technical and material support; X.C., N.C., and Q.Z. analyzed the research data and revised the manuscript. All of the authors discussed the results and approved the final manuscript.

## Declaration of interests

The authors declare no competing interests.

## STAR★Methods

### Key resources table

**Table:** 

REAGENT or RESOURCE	SOURCE	IDENTIFIER
**Antibodies**		

E2F5	ImmunoWay	Cat# YT1445; RRID: AB_3756717
AGO2	Abcam	Cat# ab186733; RRID: AB_2317978
β-actin	CST	Cat# 4967S; RRID: AB_3756722
α-tubulin	Abcam	Cat# ab52866; RRID: AB_869989
anti-IgG	Abcam	Cat# b205718; RRID: AB_3756720
antibiotin-HRP	CST	Cat# 7075; RRID: AB_3756723
Ki-67	ZSGB-BIO	Cat# ZM-0166; RRID: AB_3756724

**Bacterial and virus strains**		

LentiViral: pLV-CMV	Miaoling plasmid	Cat#BDP39198

**Chemicals, peptides, and recombinant proteins**		

TRIzol reagent	Life Technologies	Cat# 15596018
HiScript III All-in-one RT SuperMix	Vazyme	Cat# R323-01
2 × Taq Master MixVazyme	Vazyme	Cat#P111-03
TB Green® Premix Ex Taq™ II	Takara	Cat# RR420Q
Fetal bovine serum	Life Sciences	Cat#BS1612-109
DMED	Life Sciences	Cat# B1101-001
RPMI 1640	Life Sciences	Cat# B1106-022
Lipofectamine 3000	Invitrogen	Cat# L3000001
RIPA lysis buffer	Beyotime	Cat# P0013B
protease inhibitor cocktail	CWBIO	Cat# CW2200S
ProteinA/G beads	Invitrogen	Cat# 88802

**Critical commercial assays**		

PARISTM Kit	Thermo Scientific	Cat# AM1921
TranscriptAid T7 High Yield Transcription Kit	Thermo Fisher	Cat# K0441
RNA‒Protein Pull-Down Kit	Thermo Fisher	Cat# 20164
Cell Counting Kit-8	DOJINDO	Cat# TU738
CellLight EdU Apollo®567	RiboBio	Cat# C10310-1

**Experimental models: Cell lines**		

PC-3	CRL-1435	ATCC
DU145	HTB-81	ATCC
C4-2B	CRL-3315	ATCC
LNCaP	CRL-1740	ATCC
RWPE-1	CRL-3607	ATCC

**Oligonucleotides**		

ASO-SNORD116-17#1	ACCGTCATCCTCGTCGAACT	RiboBio
ASO-SNORD116-17#2	CTATACCGTCATCCTCGTCG	RiboBio
Mimic-miR206	UGGAAUGUAAGGAAGUGUGUGG	RiboBio
inhibitor-miR206	CCACACACUUCCUUACAUUCCA	RiboBio

### Experimental model and study participant details

#### Xenograft tumor model

The animal experiment was approved by the Experimental Animal Ethics Committee, West China Hospital of Sichuan University (No. 20220223073) and was performed in accordance with the guidelines by the care and use of laboratory animals. The mice were randomly divided into two groups, and DU145 cells (1×10^7^ in 150 μL PBS) were injected subcutaneously in flank of the male BALB/c nude mice (5 weeks old, *n* = 12). Tumor size was measured at 3-day intervals. When the mean volume reached 200–300 mm^3^, all the mice were euthanized to get fresh tumors and the tumor volume was calculated by (length × width^2^)/2. Then, the xenografts were analyzed by Q-PCR and IHC staining with the Ki-67 (clone number OTI8H5, ZSGB-BIO, Beijing, China). Tumor cell proliferation was assessed by the tumor volume and Ki-67 proliferation index, which was determined as the ratio of Ki-67 positive antigen staining/total number of nuclei. Because this study focuses on prostate cancer, which predominantly affects males, only male mice were utilized for xenograft modeling. Therefore, the potential influence of biological sex on tumor growth and molecular phenotypes could not be evaluated, which represents a recognized limitation of this animal experiment.

#### Cell lines

The human prostate adenocarcinoma cell lines PC-3 (CRL-1435, ATCC), DU145 (HTB-81, ATCC), C4-2B (CRL-3315, ATCC), and normal human prostate epithelial cell line LNCaP (CRL-1740, ATCC) were obtained from ATCC and maintained in RPMI 1640 medium (B1106-022, BIOEXPLORER® Life Sciences, US) with 10% fetal bovine serum (BS1612-109, BIOEXPLORER® Life Sciences, US) at 37°C with 5% CO_2_. The human normal prostate epithelial cell line RWPE-1 (CRL-3607, ATCC) was obtained from ATCC and maintained in Dulbecco’s modified Eagle’s medium (B1101-001, BIOEXPLORER® Life Sciences) supplemented with 10% fetal bovine serum (BS1612-109, BIOEXPLORER® Life Sciences) at 37°C with 5% CO_2_. All cell lines were authenticated by short tandem repeat (STR) profiling. Mycoplasma contamination was routinely examined to confirm that all cell lines were mycoplasma-free.

#### Patient tissue collection

The samples were collected from the West China Hospital of Sichuan University and included 7 cases of fresh prostate cancer (PCa) tissues and 3 cases of benign prostatic hyperplasia (BPH) tissues. All specimens were obtained with relevant ethical approval (approval number: 1703(2021)), and no patients received preoperative radiotherapy or chemotherapy before sample collection. Samples were allocated into two groups according to pathological diagnosis: the PCa group and the BPH group. All participants were male Chinese adults aged 50–69 years, with a mean age of 58 years in the BPH group and 62 years in the PCa group. Given the male-specific occurrence of prostate cancer, only male patients were enrolled; thus, the potential effects of biological sex on clinical and molecular outcomes could not be evaluated, which constitutes a limitation of this study.

### Method details

#### Ectopic expression plasmid and RNA interference (RNAi)

The pcDNA3.1-SNORD116-17 expression plasmid was constructed by inserting the complete wild-type sequence of SNORD116-17 (precursor of SNORD116-17, pWT) and the upstream exons and introns as well as the downstream introns (total 378bp). and the pcDNA3.1-SNORD116-17 mutant expression plasmid (mut) was constructed by overlapping extension PCR and sequencing. The mutant SNORD116-17 were mutated the sequences which were complementary to miR-206. The PCR primers used are listed in Table 1.

Negative control antisense oligonucleotides (ASOs) and ASOs targeting human SNORD116-17 were obtained from RiboBio (Guangzhou, China) with the following target sequences: ASO-SNORD116-17#1 (5′-ACCGTCATCCTCGTCGAACT-3′) and ASO-SNORD116-17#2 (5′-CTATACCGTCATCCTCGTCG-3′).

Mimic-miR206 (NR_029713.1), mimic-negative control (NC), inhibitor-miR206, and inhibitor-negative control (NC) were purchased from RiboBio (Guangzhou, China). The interference assays were conducted in PC-3 and DU145 cells, and the experiments were performed in triplicate.

#### Transient cell transfection, RNA extraction, nuclear-cytosol RNA extraction, reverse transcription PCR (RT‒PCR) and reverse transcription real-time quantitative PCR (Q-PCR)

PC-3 and DU145 cells were grown to 60%–70% confluence, and the respective plasmid or RNAi reagents were transfected with Lipofectamine 3000 according to the manufacturer’s protocol (L3000001, Invitrogen).

At 48 h after transfection, total RNA was extracted via TRIzol reagent (9109, TaKaRa). Nuclear-cytoplasmic RNA was extracted with a PARIS kit (AM1921, Invitrogen) according to the manufacturer’s protocol.

Reverse transcription was performed in a 20-μL volume containing 2 μg of total RNA with random primers or specific reverse primers. Standard PCR protocols were used (Vazyme, Nanjing, China). The products were resolved in a 4% agarose gel and stained with the fluorescent dye GoldView (NE006, ZHHC). Images were captured with a ChemiDoc XRS + system (Bio-Rad, USA). Quantitative real-time PCR was performed via the standard TB Green Premix Ex Taq (RR420Q, Takara) protocol with a QuantStudio 3 Real-Time PCR System (Thermo Fisher, Sweden). The genes ACTB (NM_001101.5) and U6 (NR_004394.1) were used as internal controls. The copy numbers of target genes (relative to ACTB or U6) were determined via the 2^−ΔΔCt^ method, with ΔΔCt = ΔCt_exp_ − ΔCt_con_ = (Ct_exp-target_ − Ct_exp-ACTB/U6_) − (Ct_con-target_ − Ct_con-ACTB/U6_), in which ‘exp’ represents the experimental group, ‘con’ represents the control group, and ‘target’ represents the gene of interest. The PCR primers used and the product lengths are listed in the Table. The 3′ terminal bases of the primers for detecting the SNORD116@ are located at different bases of the snoRNAs. PCR reaction will not be able to proceed because of the 3' terminal base of the primer cannot be complementary to the bases of cDNA template. So, SNORD116-3 and SNORD116-5 could not be clearly distinguished, while the other several snoRNA could be differentiated.

#### Western blot (WB) analysis

To prepare total cell lysates, in vitro-cultured cells at 72 h after transfection were digested and resuspended in RIPA lysis buffer (P0013B, Beyotime) supplemented with a protease inhibitor cocktail (CW2200S, CWBIO). After incubation on ice for 30 min and centrifugation at 12,000 rpm for 10 min at 4°C, the supernatant was collected and denatured at 100°C for 10 min.

Western blot (WB) analysis was performed via standard SDS‒PAGE. The protein concentrations of the lysates were determined via BCA assays. Typically, 10–20 μg of whole-cell lysates were separated on 10% acrylamide minigels and transferred onto 0.22 μm polyvinylidene difluoride (PVDF) membranes (Millipore, USA). The membrane was blocked for 1 h in 5% nonfat milk at room temperature, followed by overnight incubation at 4°C with the primary antibody diluted in the same blocking buffer. The primary antibodies used in this study included a polyclonal antibody against E2F5 (1:2000, YT1445, ImmunoWay), a monoclonal antibody against AGO2 (1:2000, ET1702-39, HUABIO), an antibody against β-actin (1:10000, 4967S, CST) and an antibody against α-tubulin (1:3000, ab52866, Abcam). After extensive washing, the membrane was incubated with the secondary antibody for 1 h at room temperature. After treatment with the Immobilon chemiluminescent HRP substrate (WBKLS0500, Millipore), the samples were washed and imaged with Chemidoc XRS + (Bio-Rad, USA).

#### RNA immunoprecipitation (RIP) assays

To detect whether the RNAs were associated with the AGO2 protein, the cells were homogenized via immunoprecipitation (IP) lysis buffer. The mixtures were incubated with anti-AGO2 (ab186733, Abcam, USA) or anti-IgG H&L (ab205718, Abcam, USA) and ProteinA/G beads (88802, Invitrogen) overnight at 4°C. After centrifugation and washing three times, the RNA‒protein complex-associated beads were dissolved in TRIzol, and then, the RNA was extracted, followed by PCR analysis.

#### Luciferase reporter assays

The firefly luciferase-expressing plasmids pGL3-Basic and Renilla pRL-CMV were purchased from Promega (TM033, E2261, Promega). Fragments of the wild and mutant E2F5 3′UTR (untranslated region) sequences and wild and mutant SNORD116-17 sequences were subsequently cloned and inserted into the pGL3-Promoter plasmid.

The wild E2F5 3′ UTR sequence (5′-NNNACCUUACAGUCUUCAGUUGUUNNN-3′) was prepared. The sequence with a site-directed mutation of the SNORD116-17 binding site (mutated from ACCUUACA to UGGAAUGU) was cloned and inserted into the pGL3-Promoter vector. SNORD116-17 wild-type and mutant strains were cloned as described previously and then inserted into the pGL3-promoter vector.

The cells were cultured in 24-well plates and transfected with 0.4 μg of the pGL3 reporter construct via Lipofectamine 3000 (L3000001, Invitrogen), with the pRL-CMV plasmid (0.02 μg) co-transfected as an internal control. The cells were then treated with ASO-SNROD116-17/ASO-control or the pcDNA3.1-SNORD116-17/pcDNA3.1 empty vector. Firefly luciferase (F) and Renilla luciferase (R) activities were assessed via a Spark® Multimode Microplate Reader (Tecan, Swiss). Each set of experiments included 3 replicates.

#### RNA dot blot analysis

RNAs containing wild-type and mutant SNORD116-17 were obtained *in vitro* via the TranscriptAid T7 High Yield Transcription Kit (K0441, Thermo Fisher) following a standard protocol. The wild-type and mutant SNORD116-17 were inserted into pcDNA3.1(+) which contained a T7 promoter. The mutation SNORD116-17 RNAs were mutated by design the mutation base sites on the forward primer and the amplified by PCR and sequencing. The mutation bases were not complementary with other miRNAs in human.

In total, 2 μL of the RNA mixture was spotted onto a nylon membrane (Millipore, activated by TE buffer) at different concentrations and then crosslinked by UV light. The biotin-labeled miR-206 probe (RiboBio, China) was activated at 65°C for 15 min and added to the nylon membrane (42°C, overnight). The membranes were incubated with antibiotin-HRP (7075S, CST) for 1 h and exposed to Chemidoc XRS + (Bio-Rad, USA) after treatment with the Immobilon chemiluminescent HRP substrate (WBKLS0500, Millipore).

#### RNA-protein pulldown assays

The biotinylated sense and antisense miR-206 RNAs were purchased from Sangon (Shanghai, China), and the biotinylated wild-type and mutation SNORD116-17 RNAs were synthesized using aforementioned TranscriptAid T7 High Yield Transcription Kit. These RNAs were incubated with PC-3 or DU145 cell extracts, followed by RNA-protein pulldown assays performed with the Pierce^TM^ Magnetic RNA-Protein Pull-Down Kit (20164, Thermo Fisher). The retrieved proteins were subsequently analyzed via Western blot analysis.

#### *In vitro* proliferation assays

Cell viability was evaluated after 0 h, 24 h, 48 h, 72 h, and 96 h of incubation by the Cell Counting Kit-8 (CCK-8; TU738, DOJINDO). The CCK-8 assays were performed via a standard protocol, and the optical density was measured at 450 nm via a spectrophotometer. The optical density at 450 nm (OD450) indicates the metabolism status and the proliferation rate of the cells. Each set of experiments included 5 replicates.

The 5-ethynyl-2′-deoxyuridine (EdU) incorporation assay was performed with a CellLight EdU Apollo®567 *In Vitro* Imaging Kit (C10310-1, RiboBio) according to the manufacturer’s instructions.

#### *In vitro* wound healing assays

Prostate cancer cells (PC-3 and DU145) were seeded in 6-well plates, and when the cells reached a density of approximately 95%–100%, an identical wound was made across the center of each well with a 200 μL plastic pipette tip. Then, the cells were transfected with ASOs or plasmids and cultivated in the medium supplemented with 2% FBS. At 24 h (DU145) or 48 h (PC-3) after wounding, the wounds were photographed (Olympus CKX53SF inverted microscope, Japan).

#### *In vitro* transwell migration and invasion assays

Cell migration and invasion assays were performed via 8 μm pore size 24-well transwell chambers (Millipore, USA) with membranes uncoated (for migration) or coated (for invasion) with Matrigel (Corning, USA); 1× 10^5^ cancer cells in 1640 medium with 0.1% FBS were placed in the upper chamber. The bottom chamber was filled with medium containing 10% FBS. After 24 h (migration) or 48 h (invasion), the cells that migrated to or invaded the lower chambers were fixed with 4% paraformaldehyde, stained with crystal violet, and then photographed (Olympus CKX53SF inverted microscope, Japan).

### Quantification and statistical analysis

Statistical analysis was carried out via SPSS 25.0 and GraphPad Prism 8. Analysis of variance (ANOVA) and Student’s t-tests or Fisher exact were applied to determine the significance of between-group comparisons, and Spearman rank correlation were used for correlation analysis, Kaplan-Meier survival analysis was used to calculate overall survival (OS) rates of patients, related data was obtained from GEO Dataset GSE16560, GSE14814, GSE26712, GSE13213, GSE9893, and GSE14333. A *p* value <0.05 was considered statistically significant. Statistical significance was defined as *p* < 0.05 (∗), *p* < 0.01 (∗∗), and *p* < 0.001 (∗∗∗).

## Supplemental information

Data S1. Raw data-uncropped blots
