# Supplementary material for: Box C/D SNORD116-17 promotes prostate cancer progression by targeting the miR-206/E2F5 axis
Source: iScience. 2026 Oct 8;29(11):117806. doi: 10.1016/j.isci.2026.117806 (PMC13677326; doi:10.1016/j.isci.2026.117806)
Supplement: Supplementary file 1 [file mmc1.pdf]

iScience, Volume *29*

Supplemental information

Box C/D SNORD116-17 promotes prostate cancer progression by targeting the miR-206/E2F5 axis

Xiuyi Pan, Yifan Kang, Jinjing Zhong, Ting Yu, Linmao Zheng, Yuyan Wei, Junya Tan, Ling Nie, Mengni Zhang, Ni Chen, Qiao Zhou, and Xueqin Chen

Data S1: Raw data-uncropped blots

FIG 1B

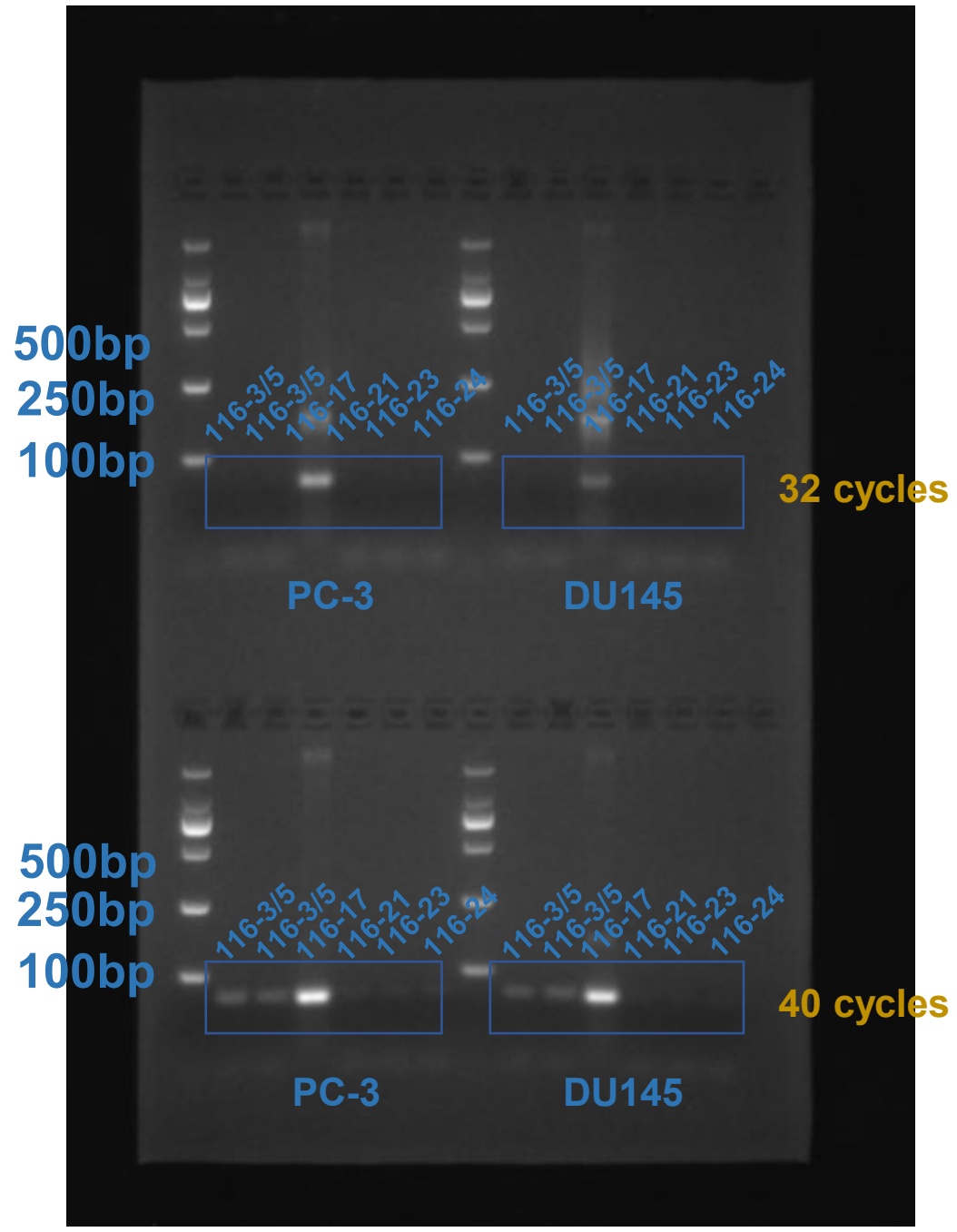

up：32 cycles amplification
lower：40 cycles amplification
left：PC-3 cells
right：DU145 cells

lane1：SNORD116-3（79bp）
lane2：SNORD116-5（79bp）
lane3：SNORD116-17（79bp）
lane4：SNORD116-21(81BP)
lane5：SNORD116-23(83BP)
lane6：SNORD116-24(85BP)

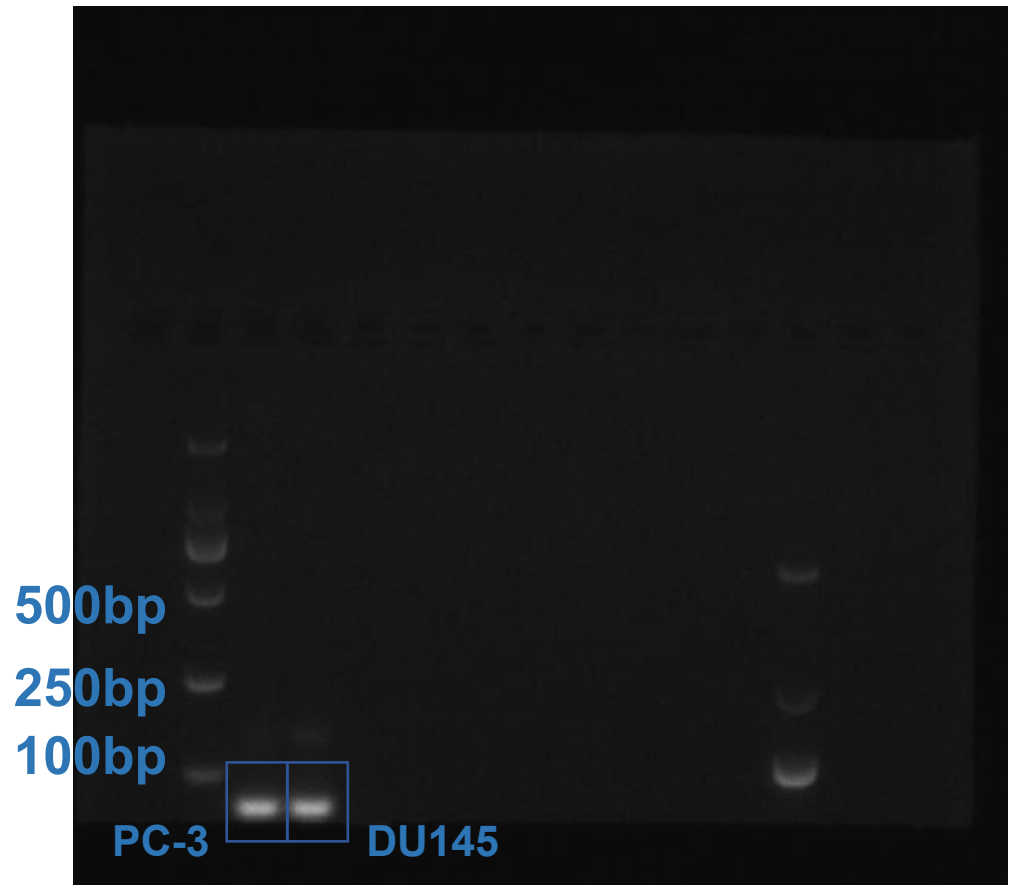

U6 gene-66bp

lane1：PC-3 cells
lane2：DU145 cells

FIG 1D

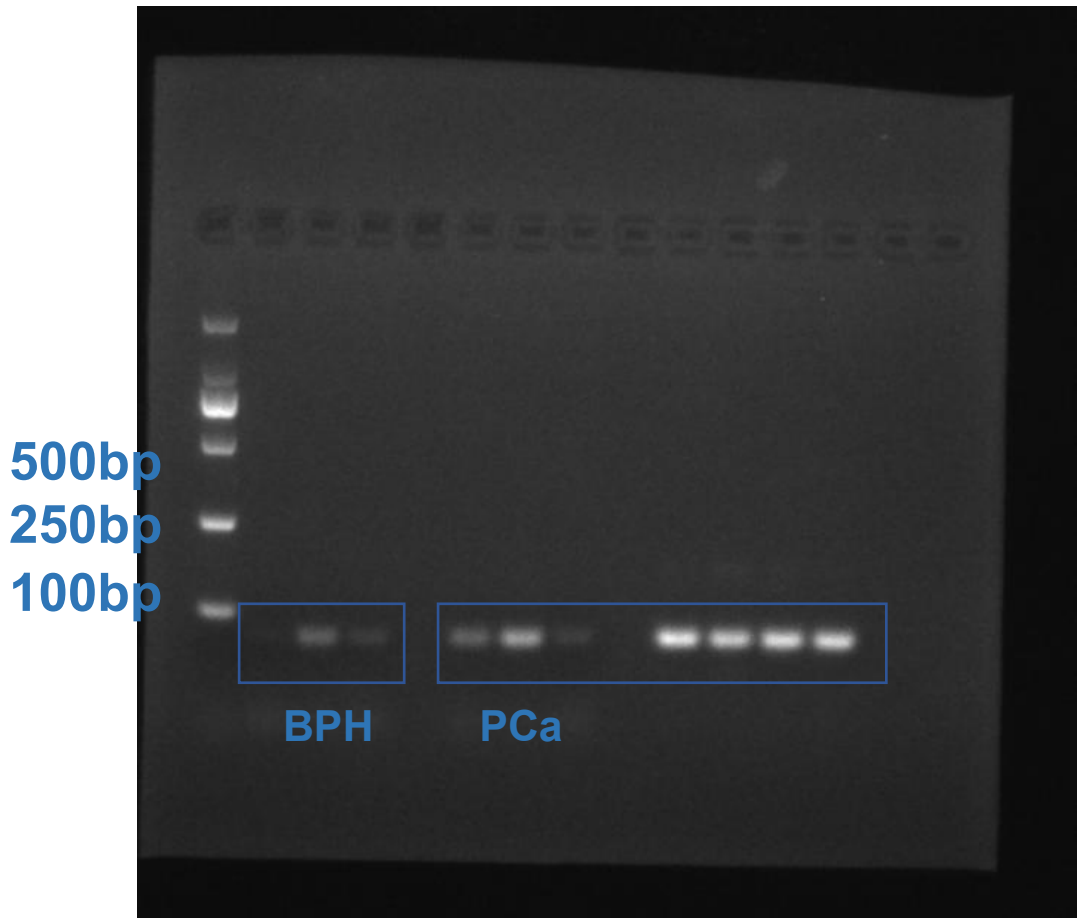

SNORD116-17 gene-primer1(79bp)
lane1-3：BPH
lane4-6：PCa

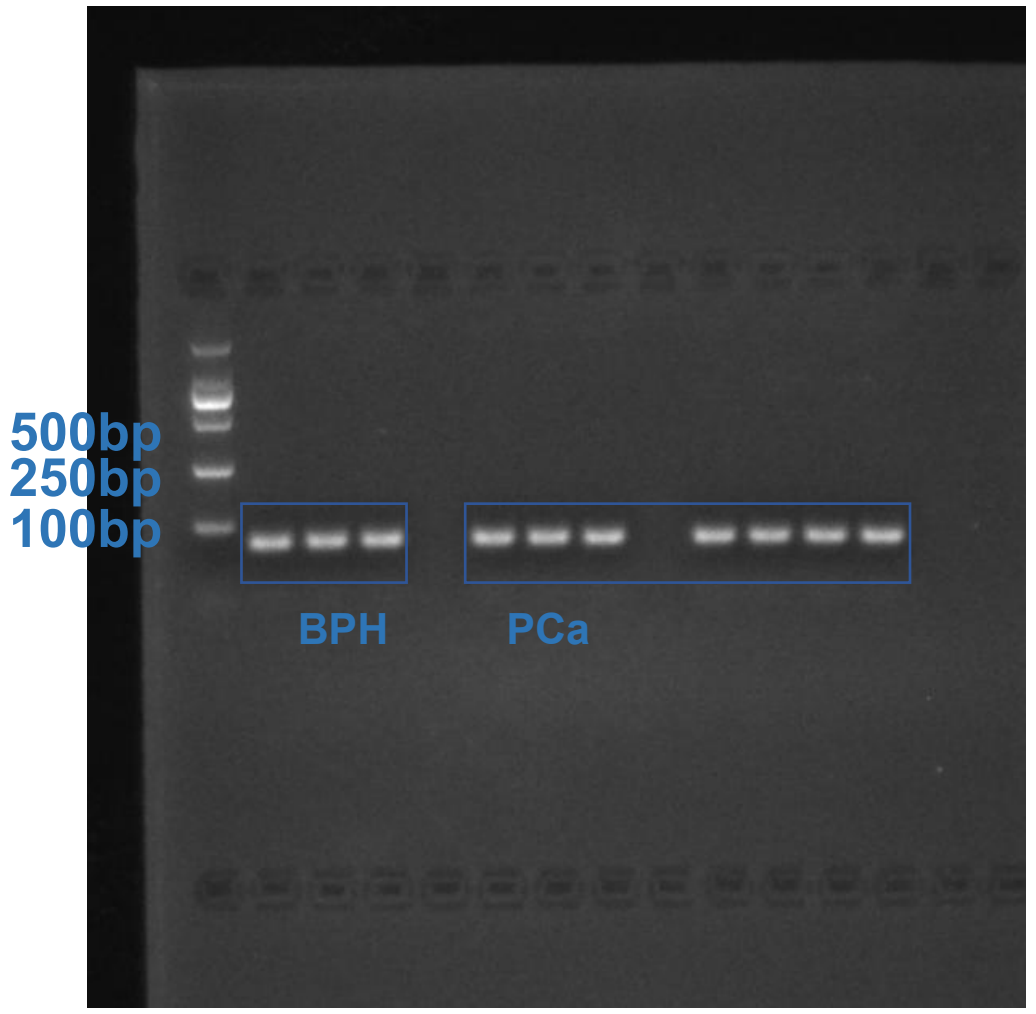

U6 gene-66bp
lane1-3：BPH
lane4-6：PCa

FID 1C

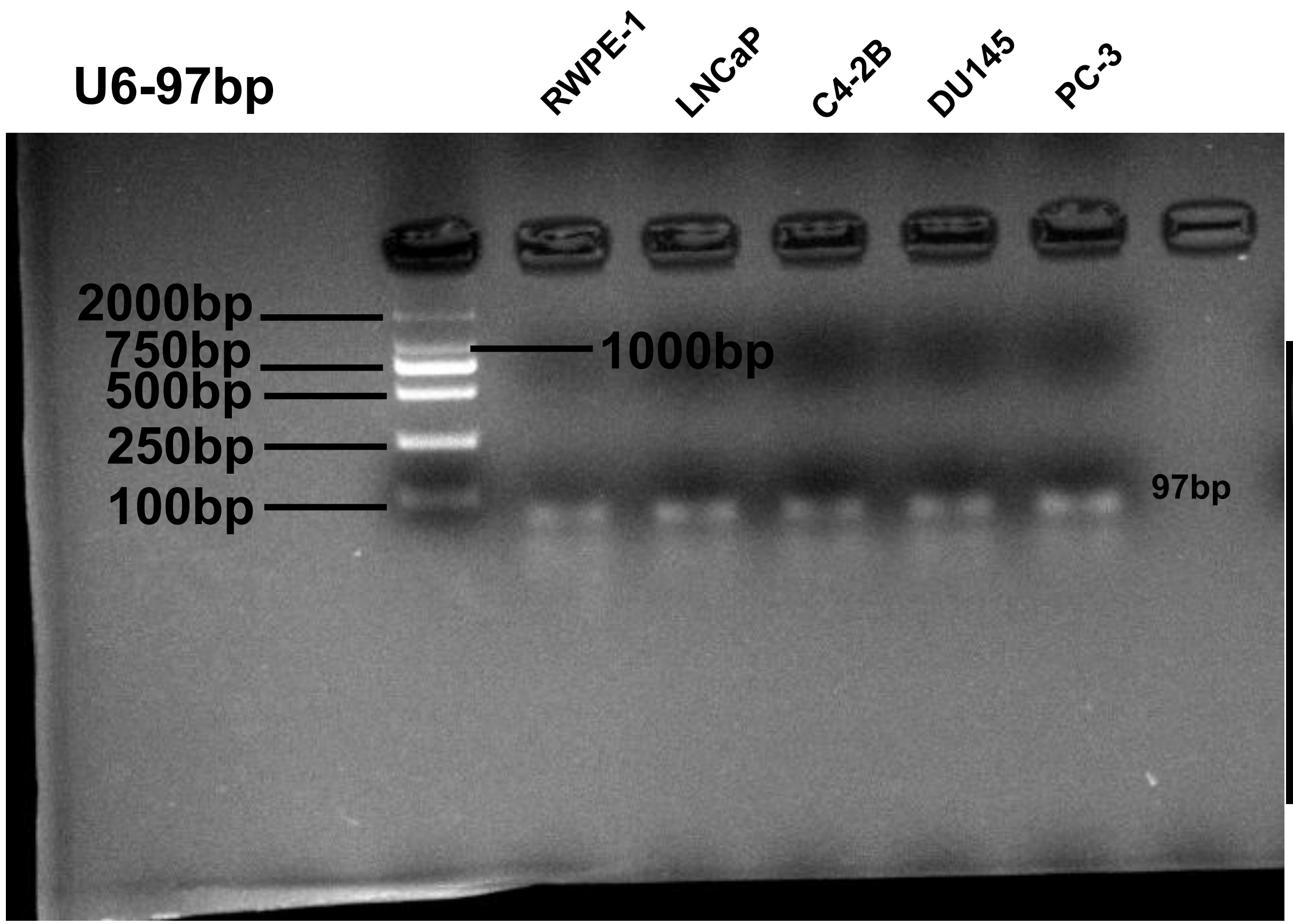

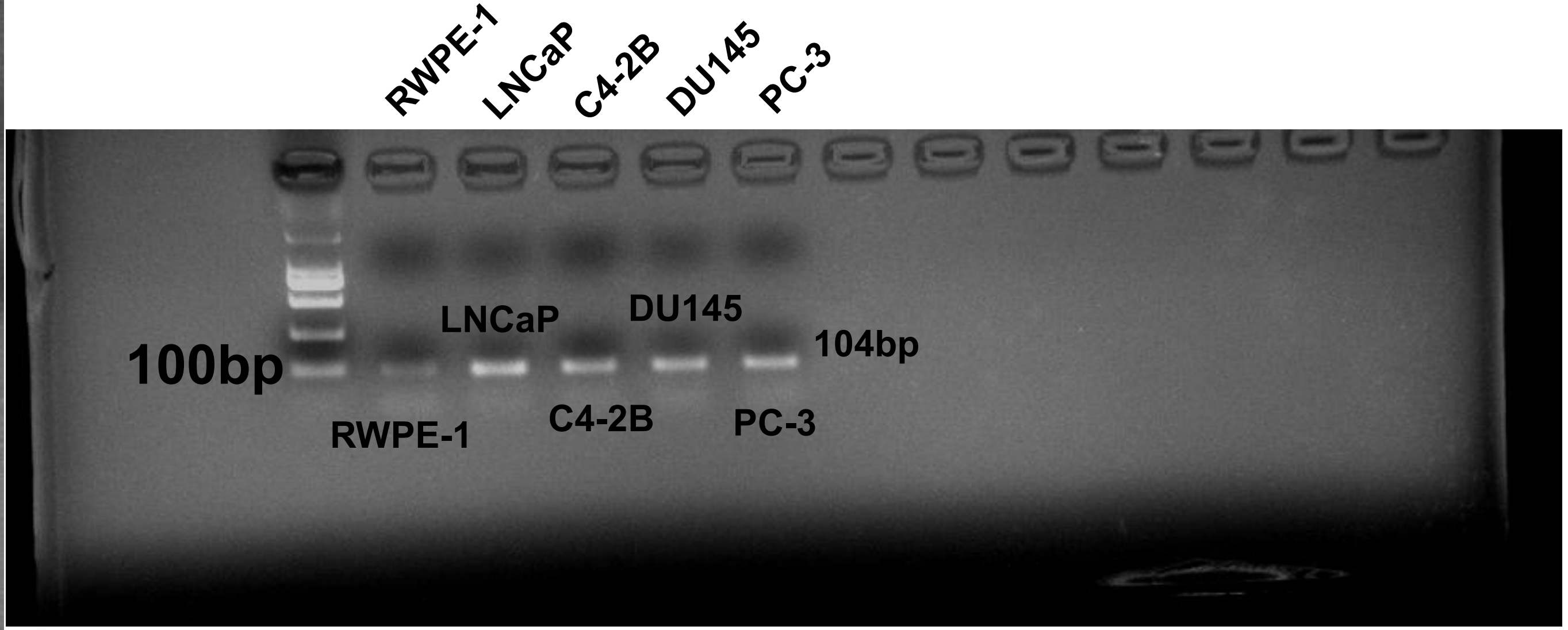

SNORD116-17-primer2（104bp）

SNORD116-17

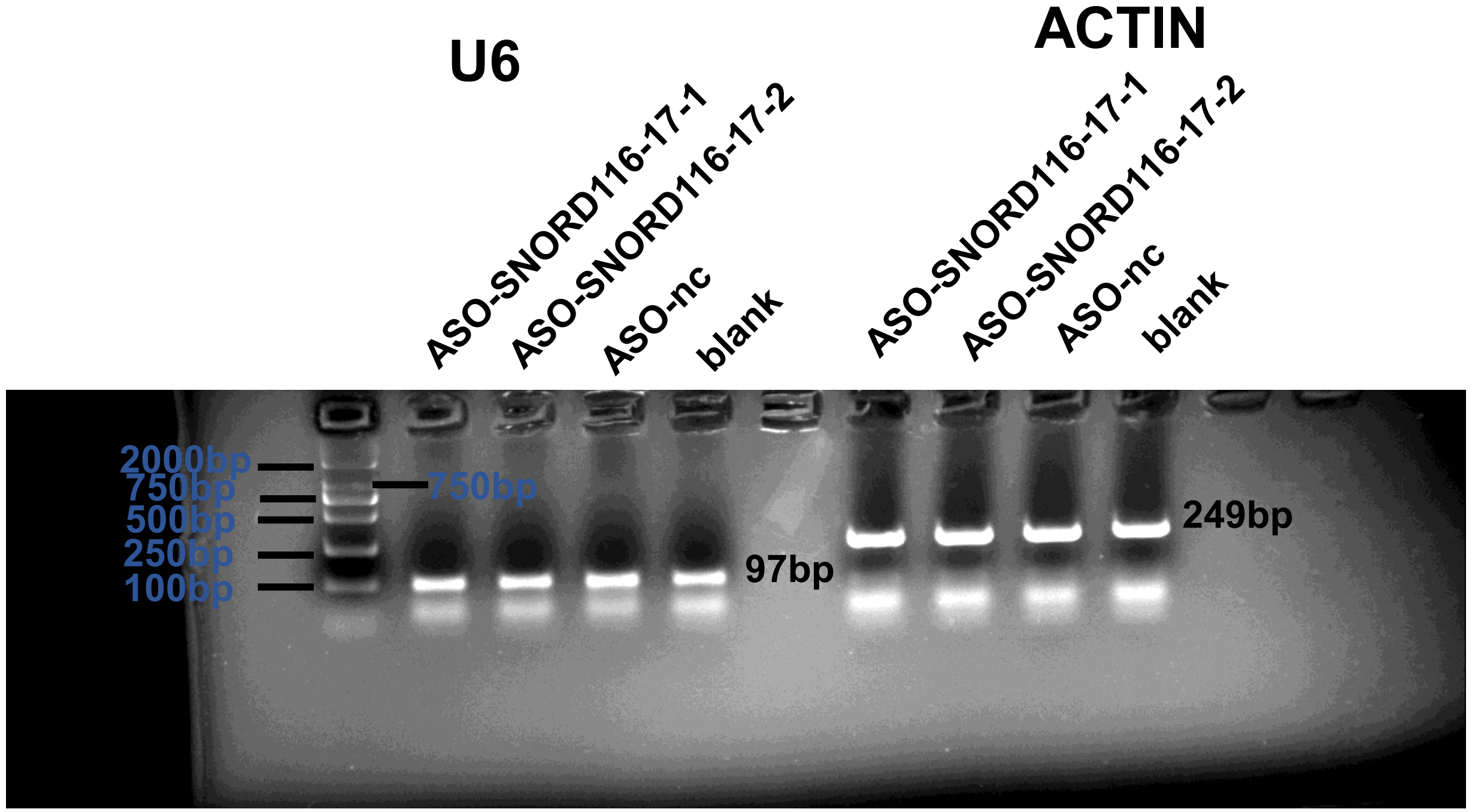
U6
ACTIN
ASO-SNORD116-17-1
ASO-SNORD116-17-2
ASO-nc
blank
ASO-SNORD116-17-1
ASO-SNORD116-17-2
ASO-nc
blank
2000bp
750bp
500bp
250bp
100bp
750bp
97bp
249bp

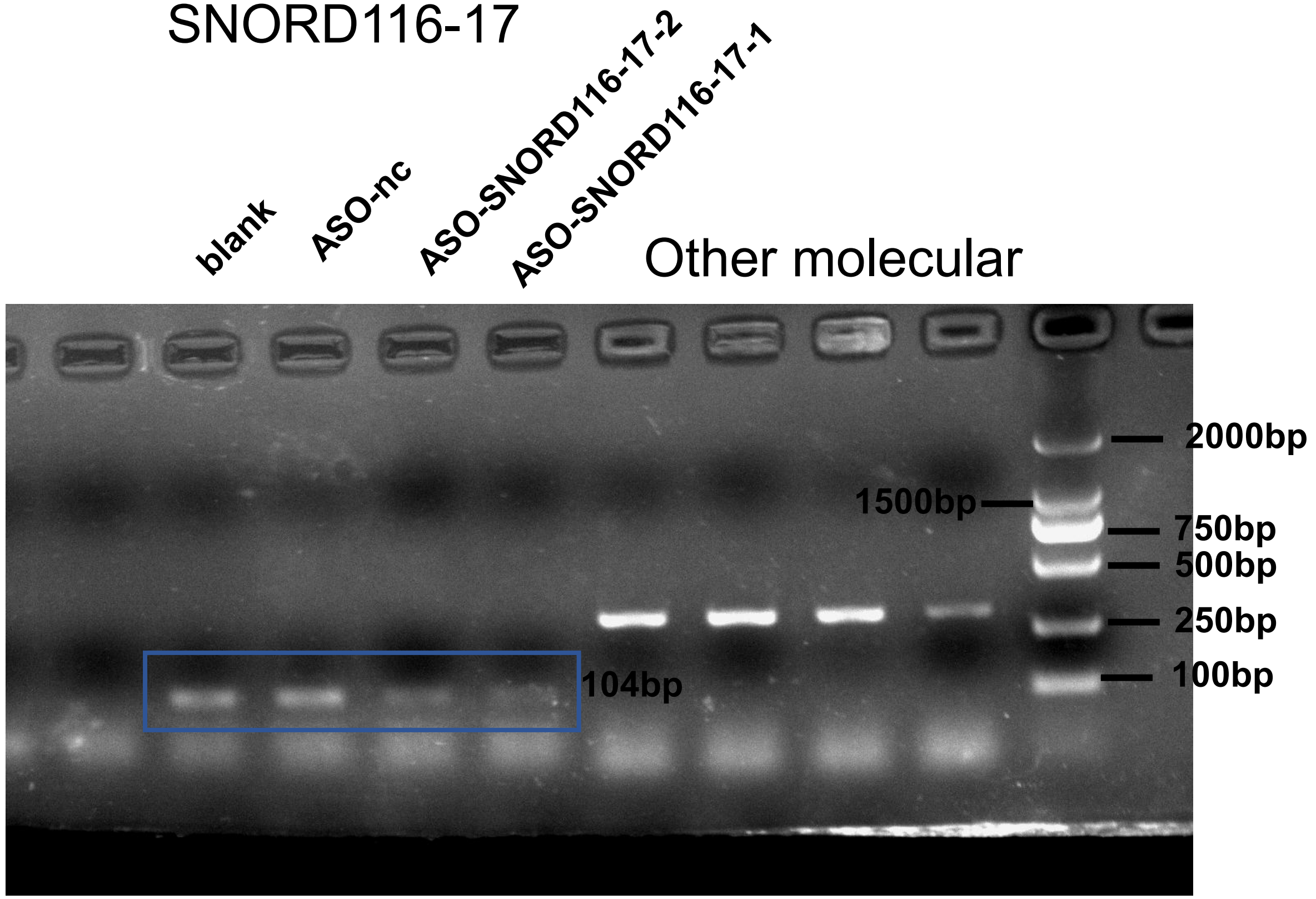
SNORD116-17
blank
ASO-nc
ASO-SNORD116-17-2
ASO-SNORD116-17-1
Other molecular
2000bp
1500bp
750bp
500bp
250bp
100bp
104bp

FIG 2A

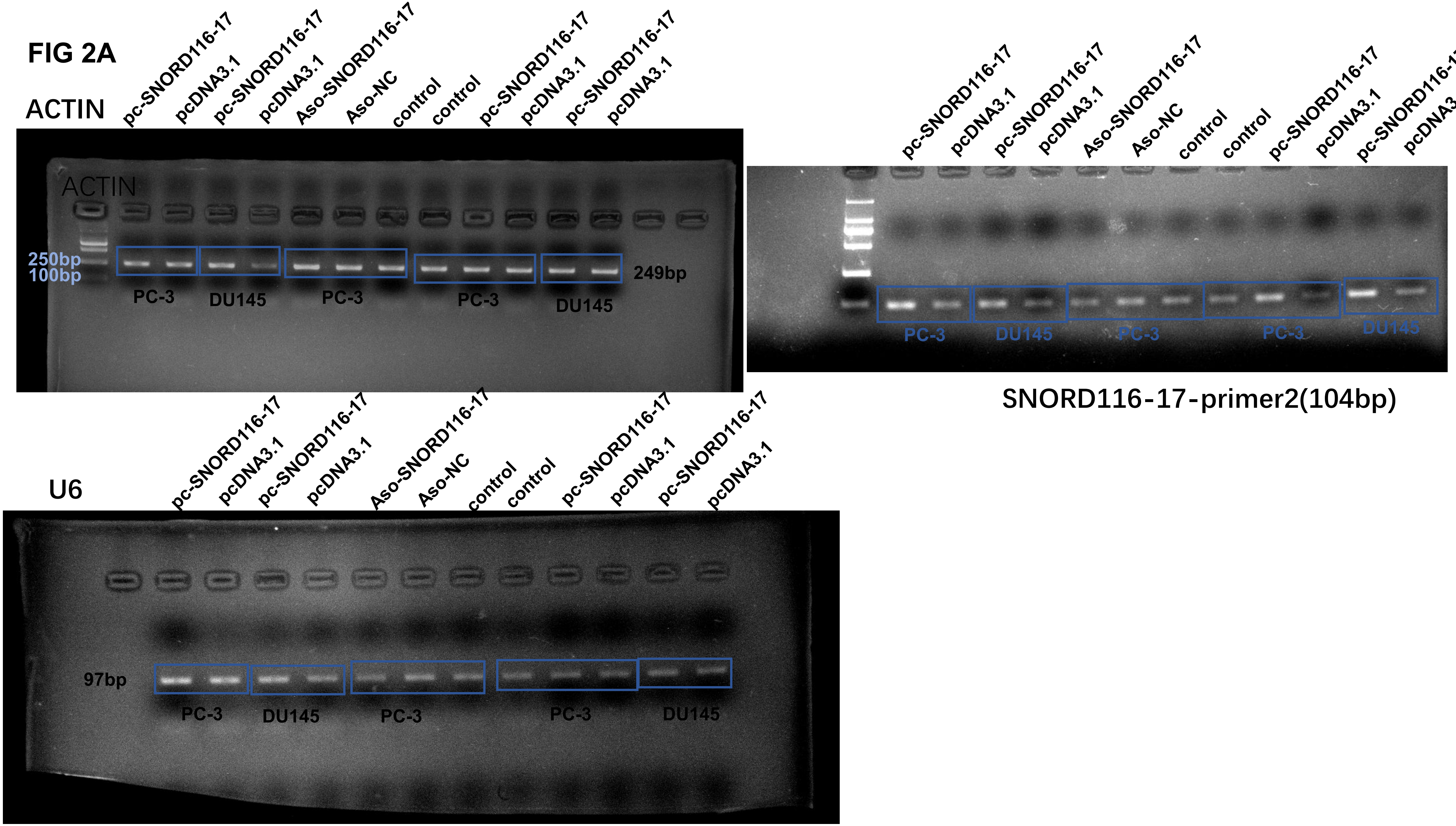
ACTIN
pc-SNORD116-17
pcDNA3.1
pc-SNORD116-17
pcDNA3.1
Aso-SNORD116-17
Aso-NC
control
control
pc-SNORD116-17
pcDNA3.1
pc-SNORD116-17
pcDNA3.1
ACTIN
250bp
100bp
249bp
PC-3
DU145
PC-3
PC-3
DU145
pc-SNORD116-17
pcDNA3.1
pc-SNORD116-17
pcDNA3.1
Aso-SNORD116-17
Aso-NC
control
control
pc-SNORD116-17
pcDNA3.1
pc-SNORD116-17
pcDNA3.1
PC-3
DU145
PC-3
PC-3
DU145
SNORD116-17-primer2(104bp)
U6
pc-SNORD116-17
pcDNA3.1
pc-SNORD116-17
pcDNA3.1
Aso-SNORD116-17
Aso-NC
control
control
pc-SNORD116-17
pcDNA3.1
pc-SNORD116-17
pcDNA3.1
97bp
PC-3
DU145
PC-3
PC-3
DU145

FIG 2A

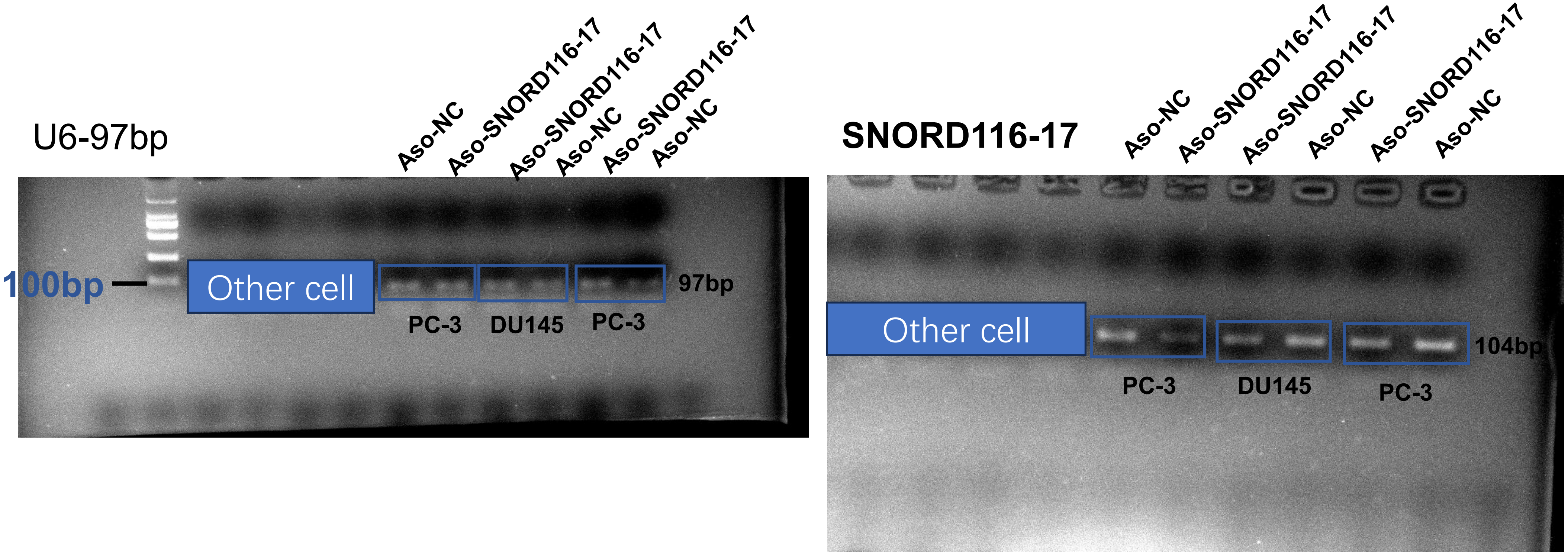

U6-97bp
Aso-NC
Aso-SNORD116-17
Aso-SNORD116-17
Aso-NC
Aso-SNORD116-17
Aso-NC
100bp
Other cell
PC-3
DU145
PC-3
97bp
SNORD116-17
Aso-NC
Aso-SNORD116-17
Aso-SNORD116-17
Aso-NC
Aso-SNORD116-17
Aso-NC
Other cell
PC-3
DU145
PC-3
104bp

PCR carried out using different primers at different temperatures

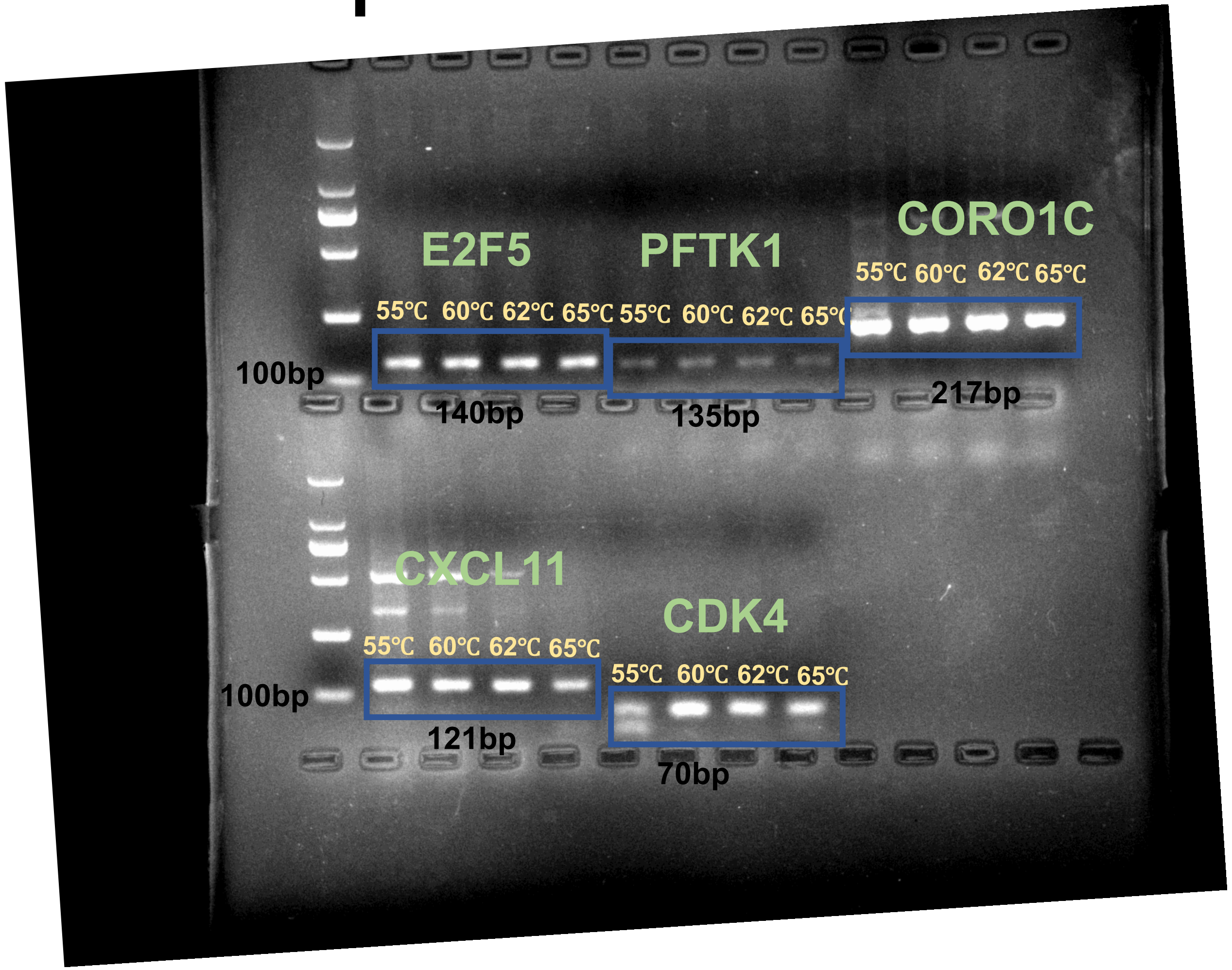

FIG 3H

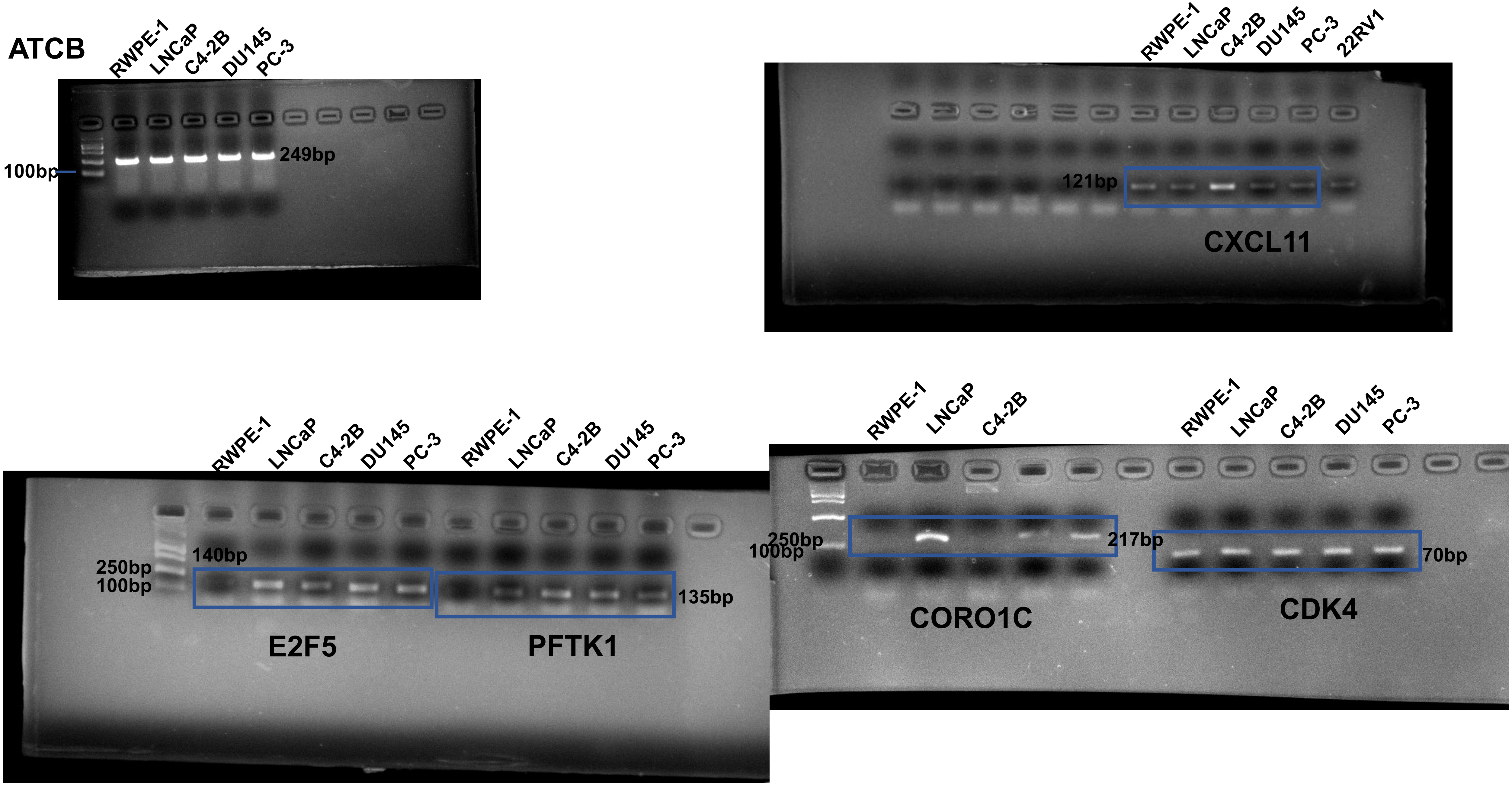
ATCB
RWPE-1
LNCaP
C4-2B
DU145
PC-3
100bp
249bp
RWPE-1
LNCaP
C4-2B
DU145
PC-3
22RV1
121bp
CXCL11
RWPE-1
LNCaP
C4-2B
DU145
PC-3
RWPE-1
LNCaP
C4-2B
DU145
PC-3
140bp
250bp
100bp
135bp
E2F5
PFTK1
RWPE-1
LNCaP
C4-2B
RWPE-1
LNCaP
C4-2B
DU145
PC-3
250bp
100bp
217bp
70bp
CORO1C
CDK4

FIG 3H

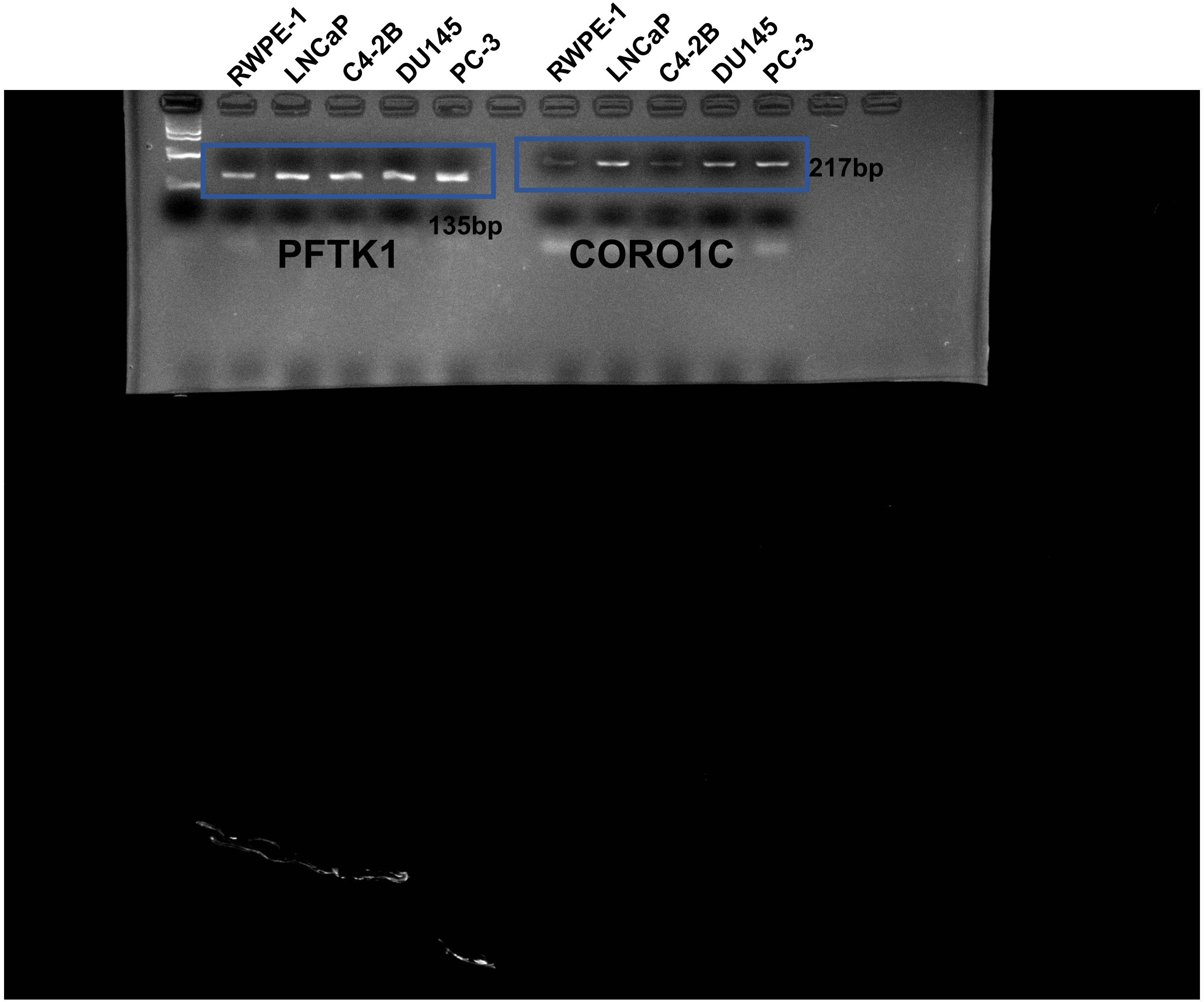

RWPE-1
LNCaP
C4-2B
DU145
PC-3
RWPE-1
LNCaP
C4-2B
DU145
PC-3
217bp
135bp
PFTK1
CORO1C

FIG 3J

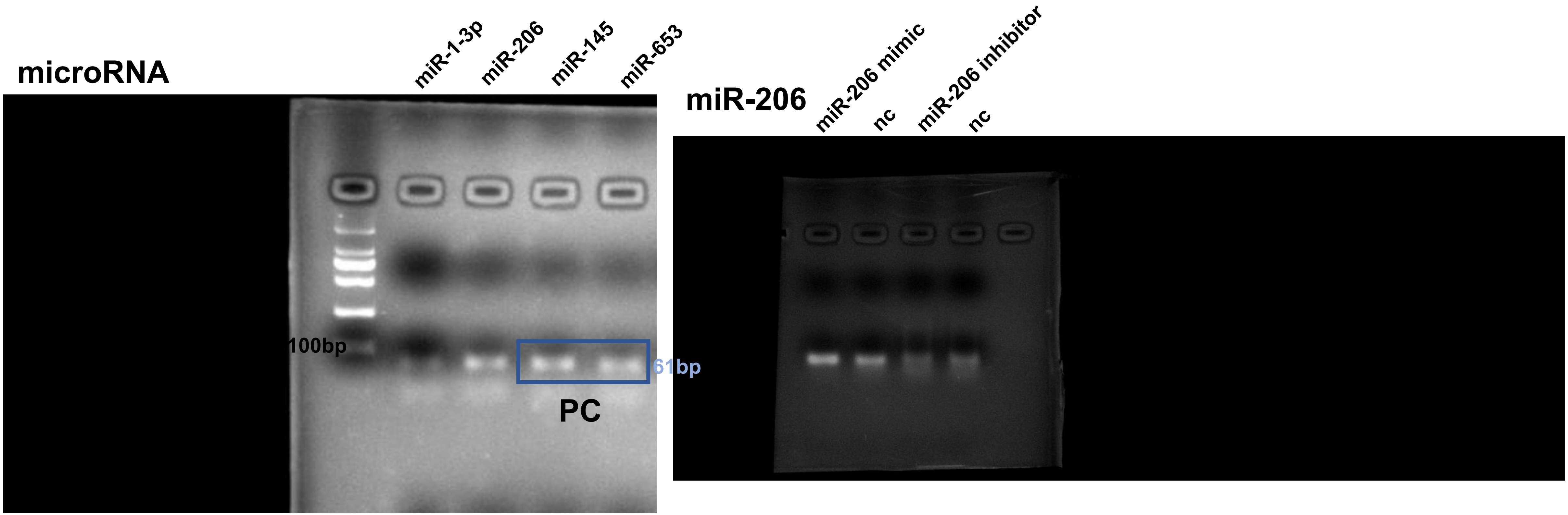
microRNA
miR-1-3p
miR-206
miR-145
miR-653
100bp
61bp
PC
miR-206
miR-206 mimic
nc
miR-206 inhibitor
nc

FIG 3J-E2F5 mRNA

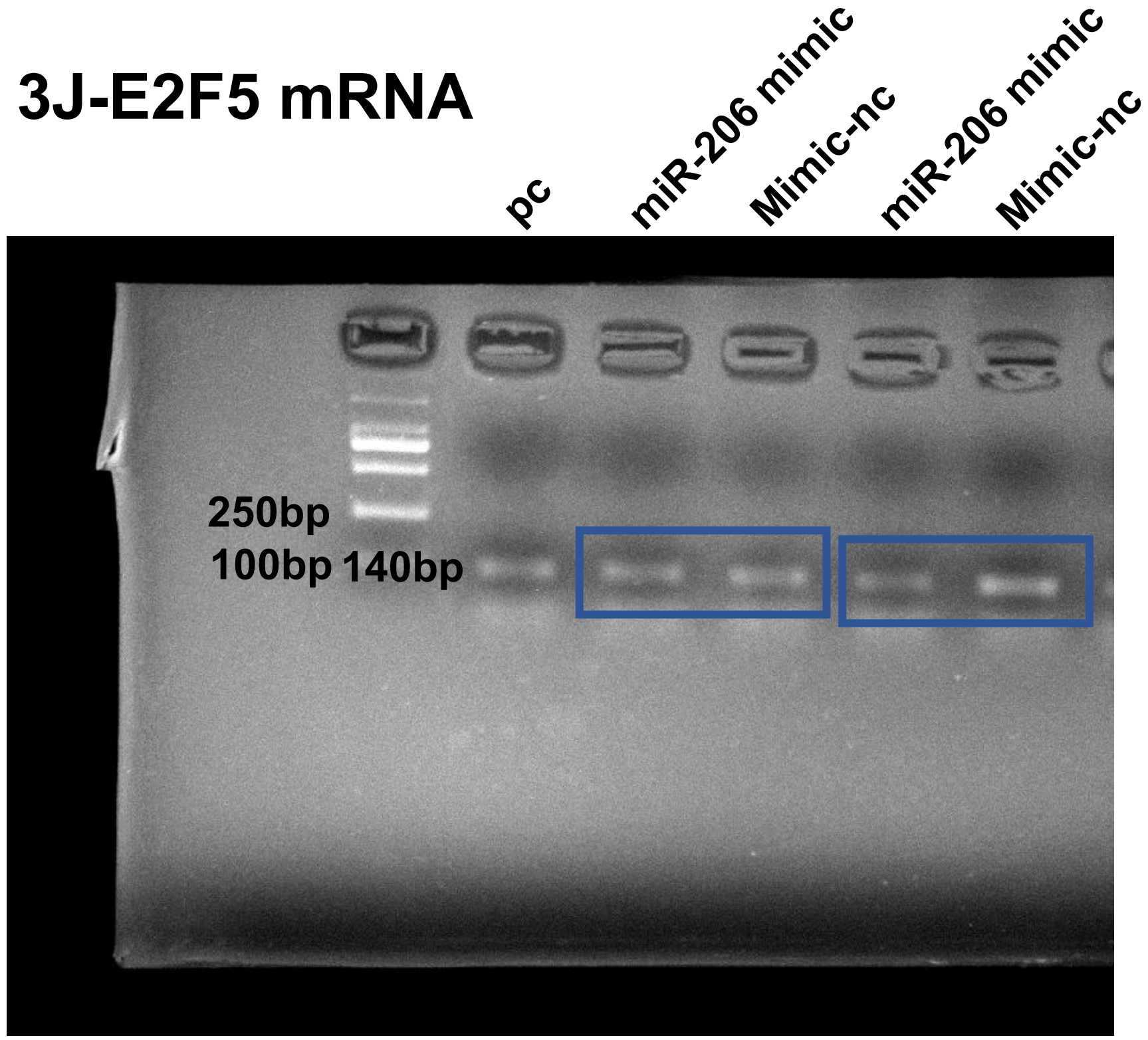

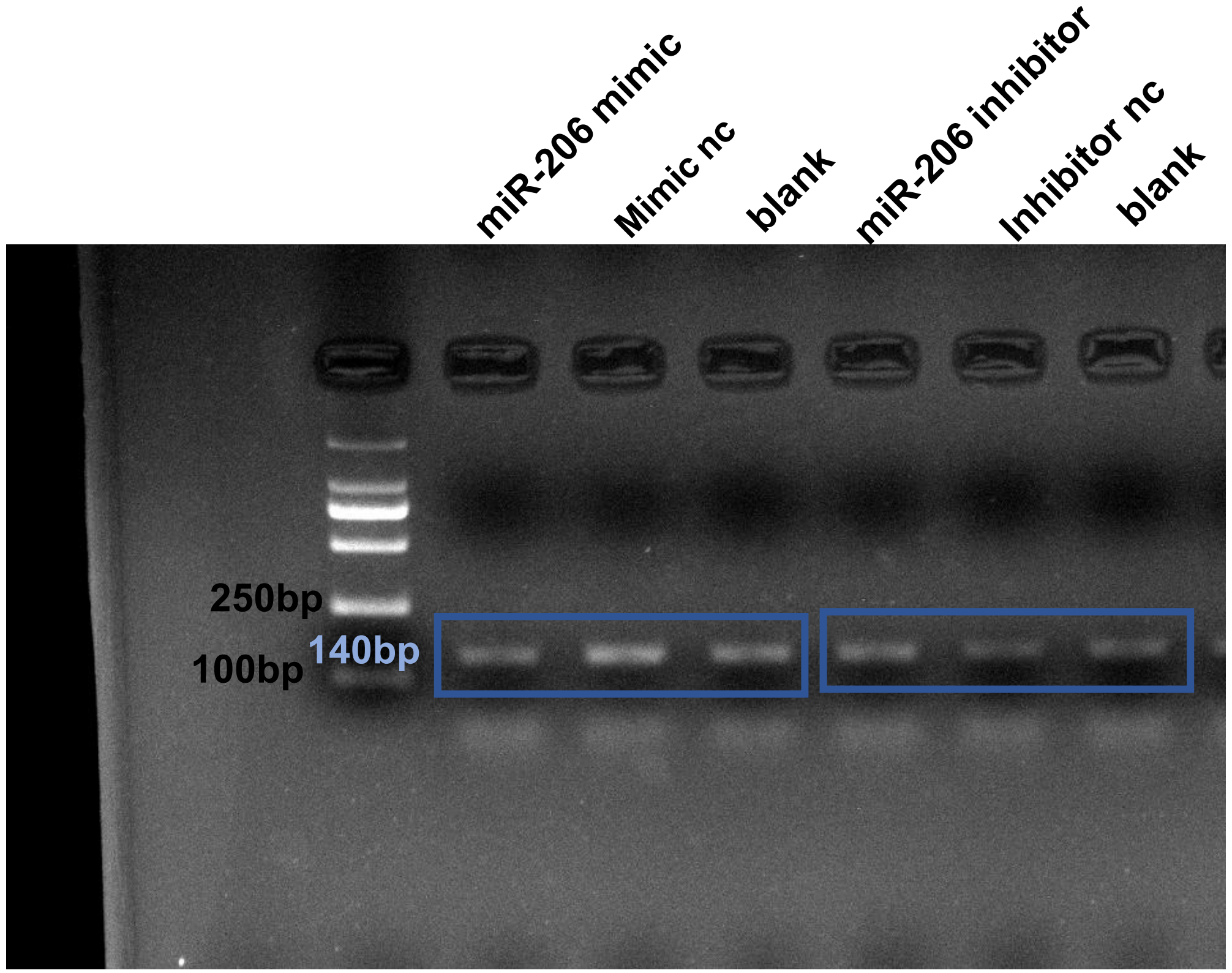

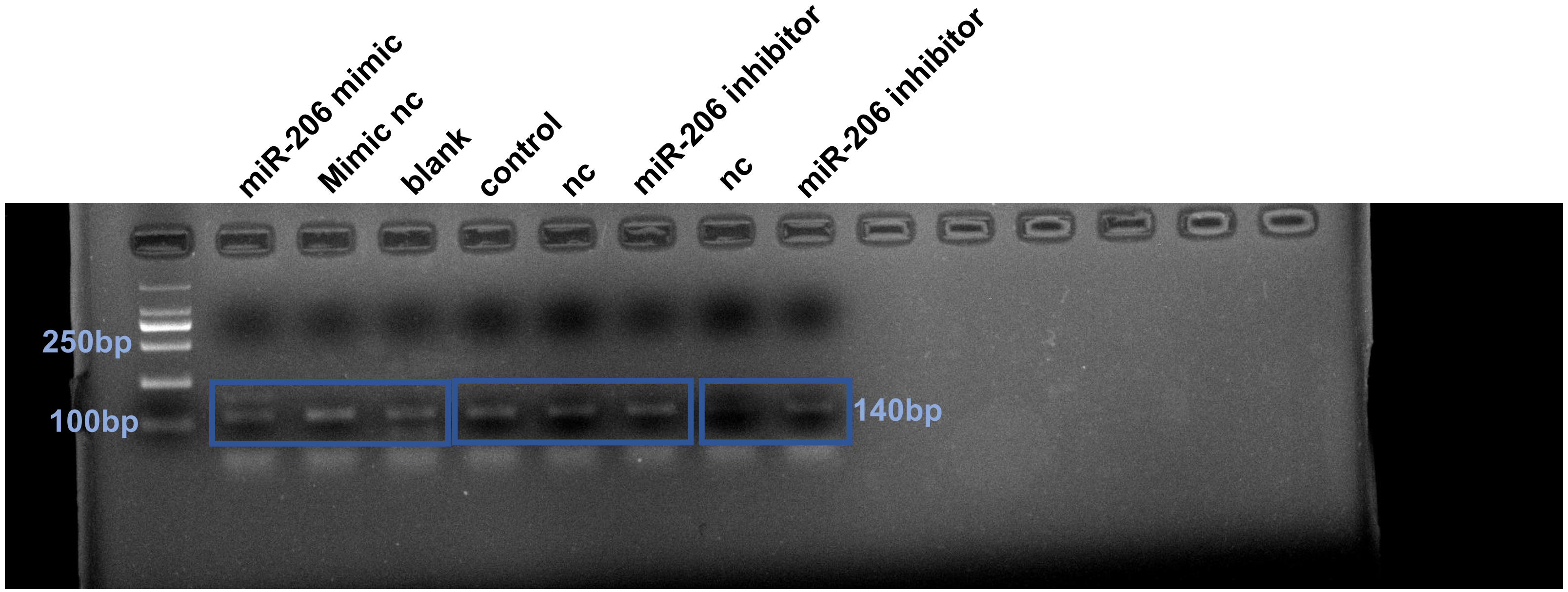

FIG 3J

CORO1C

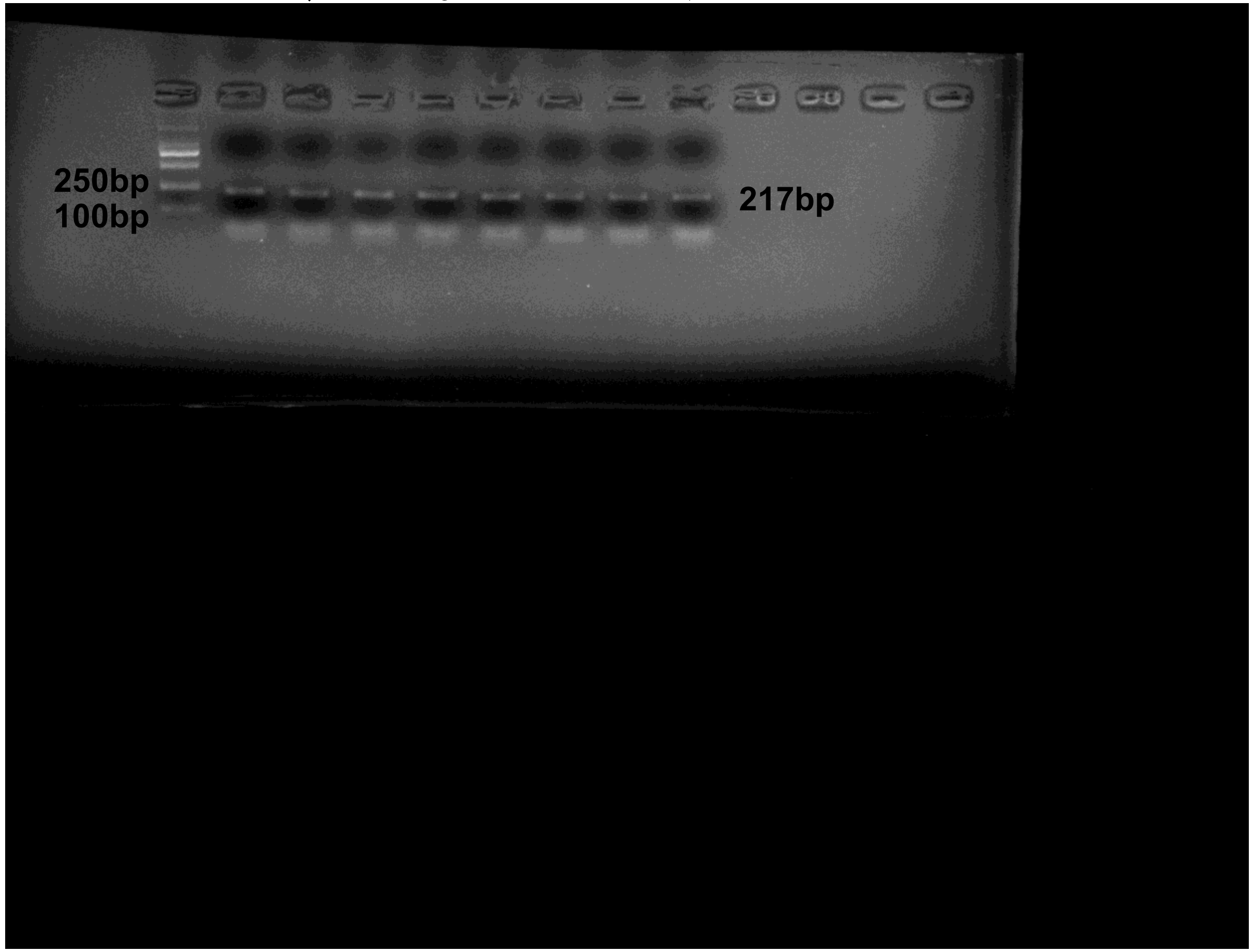

ACTIN

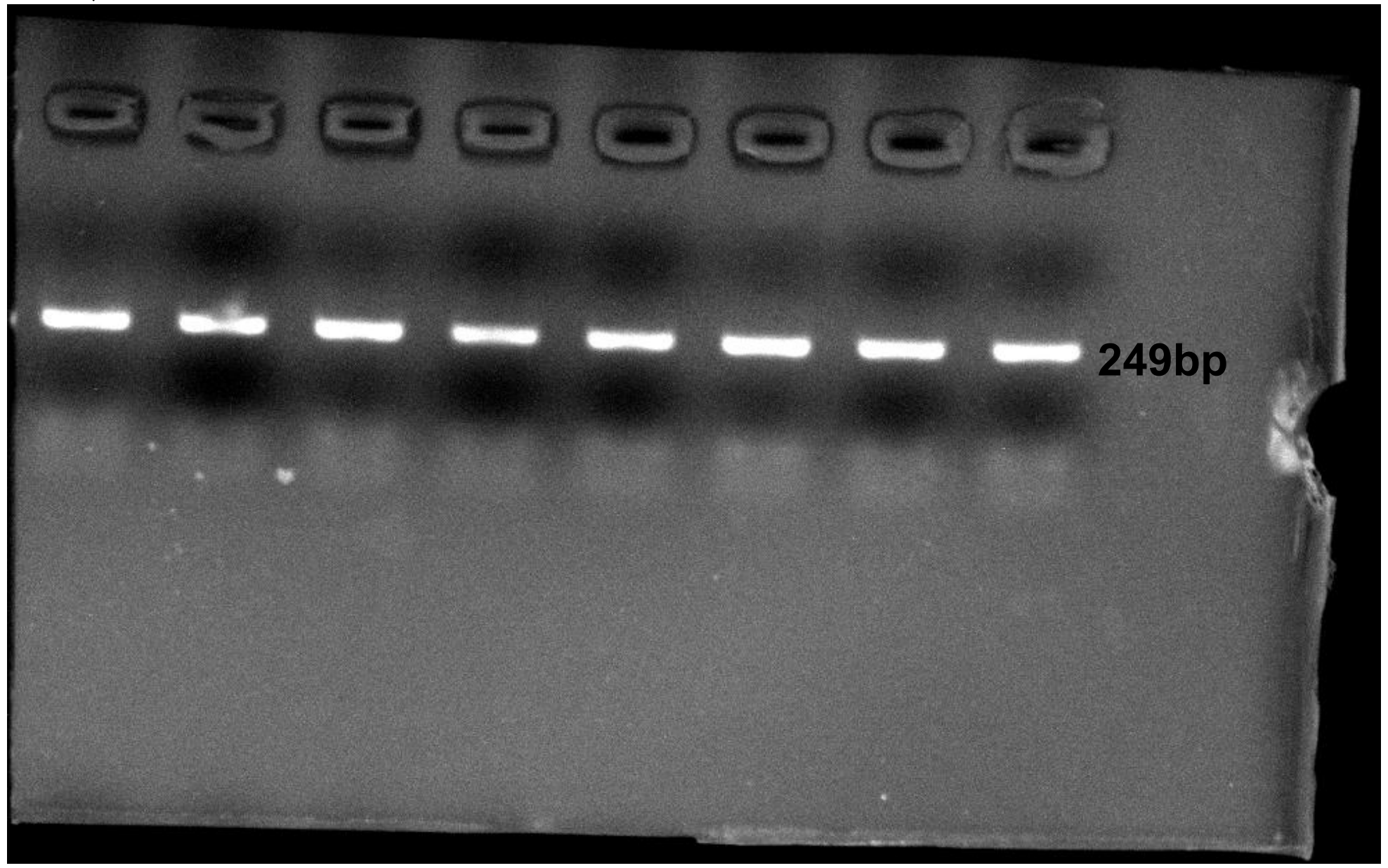

FIG 3E RIP assays-miR-206

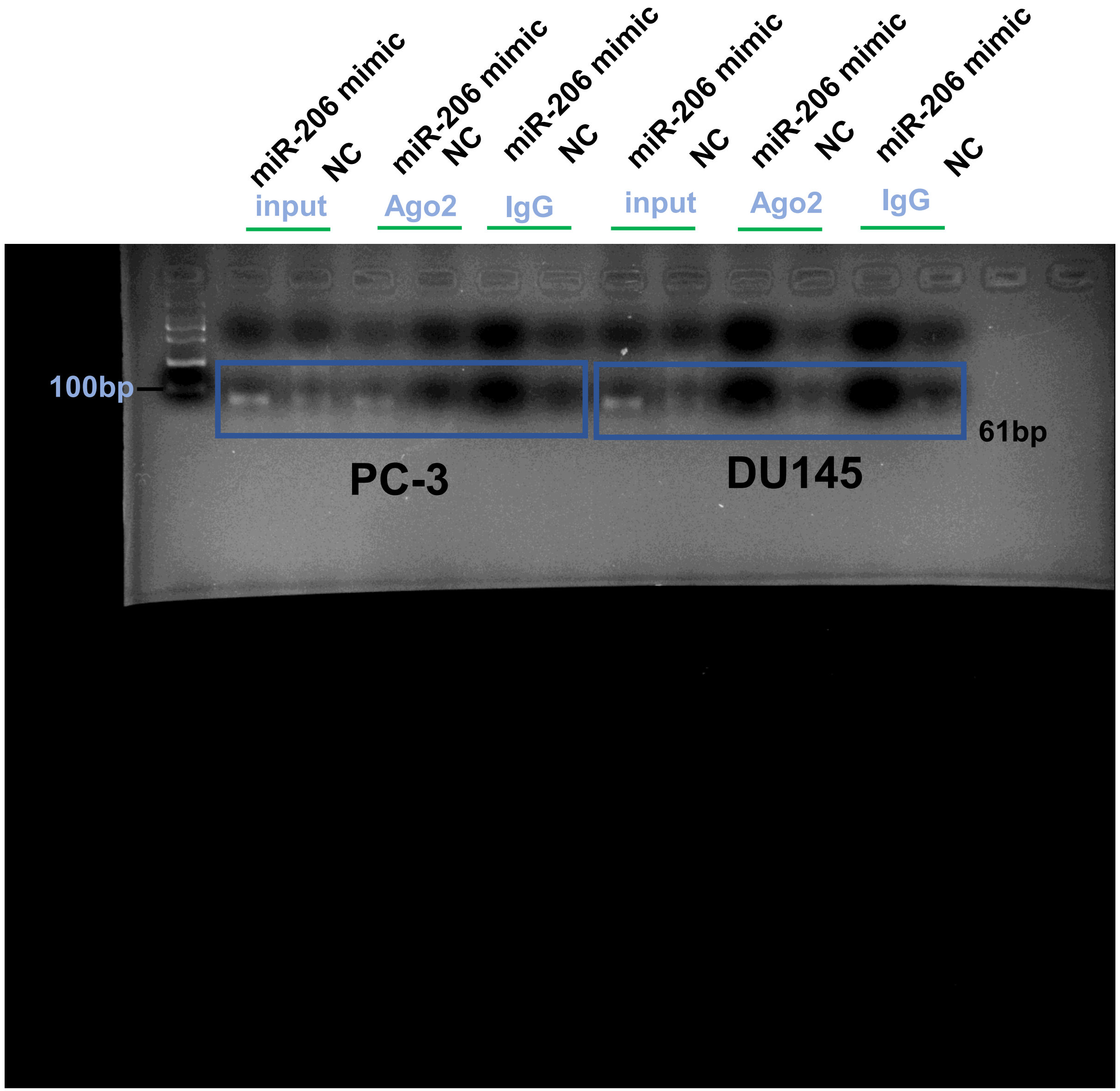

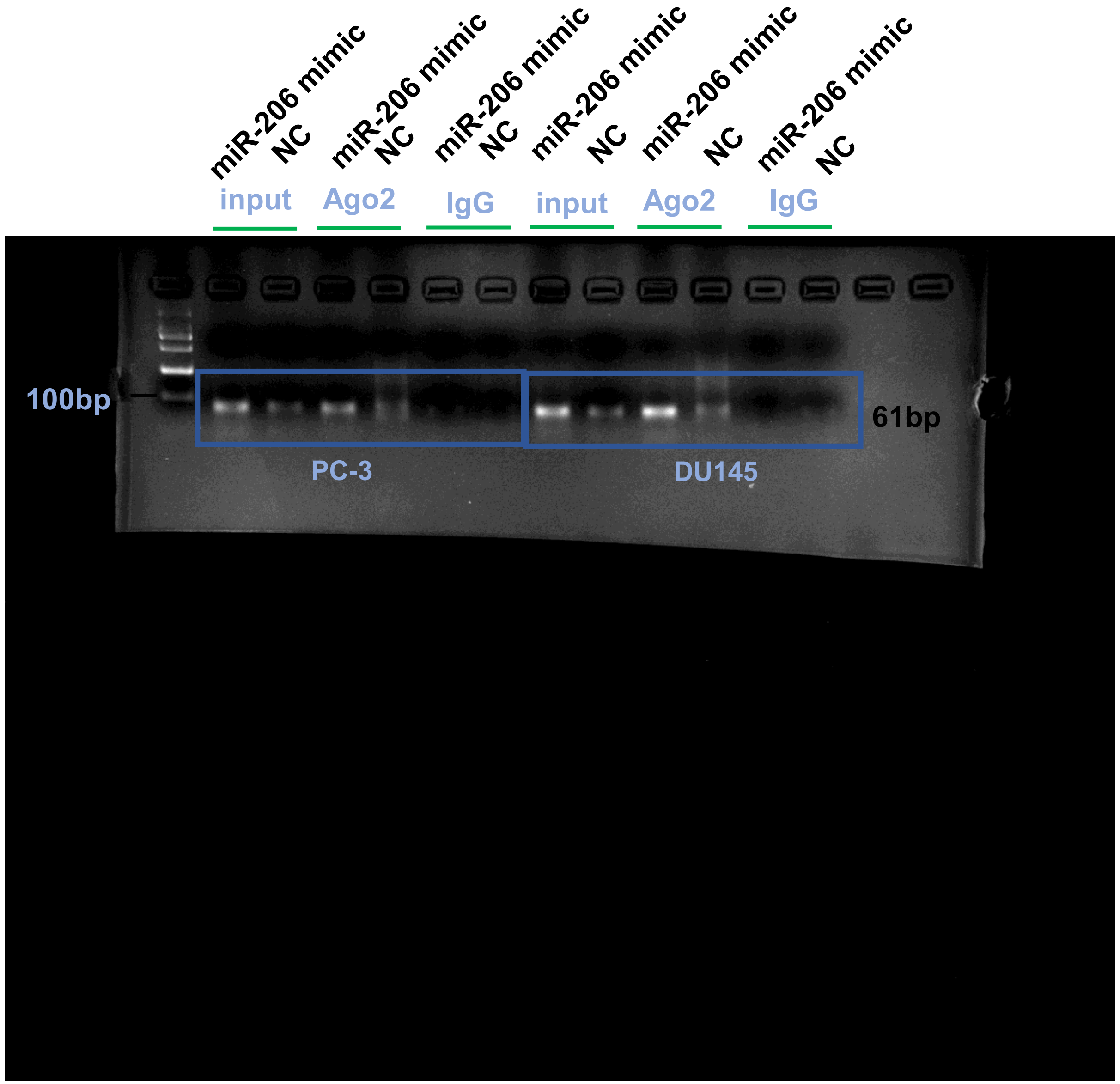

FIG 3E RIP assay SNORD116-17

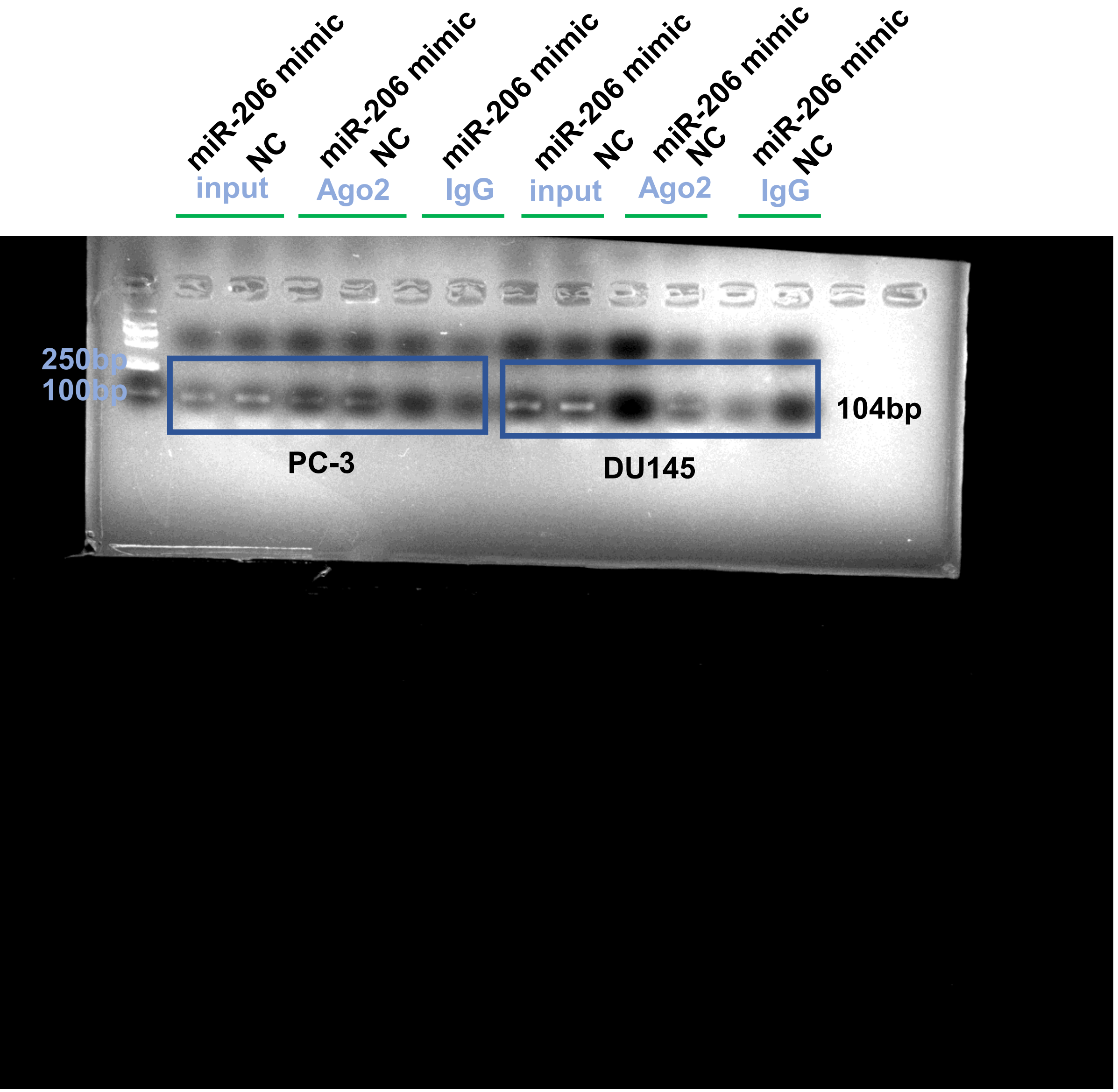

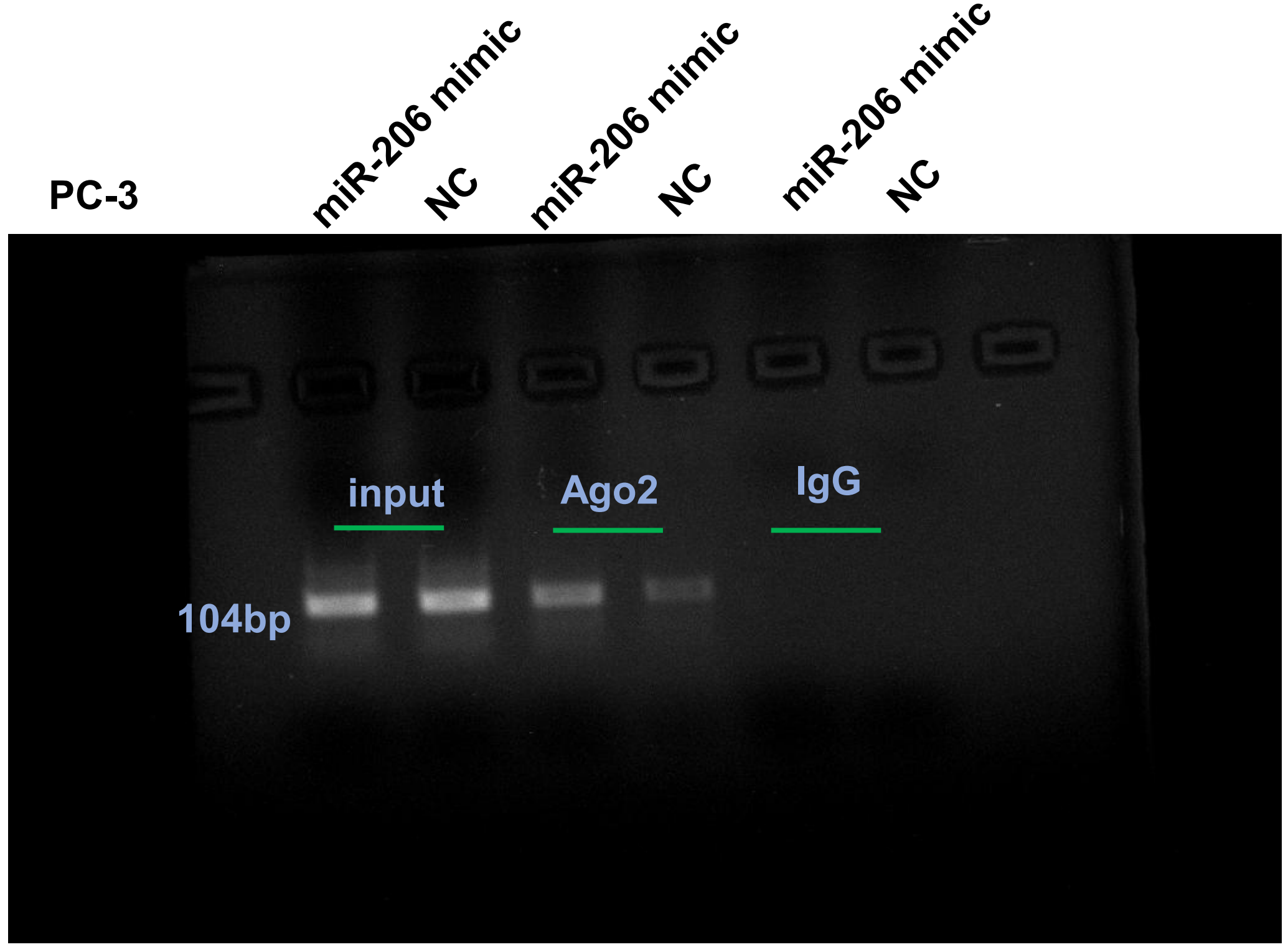

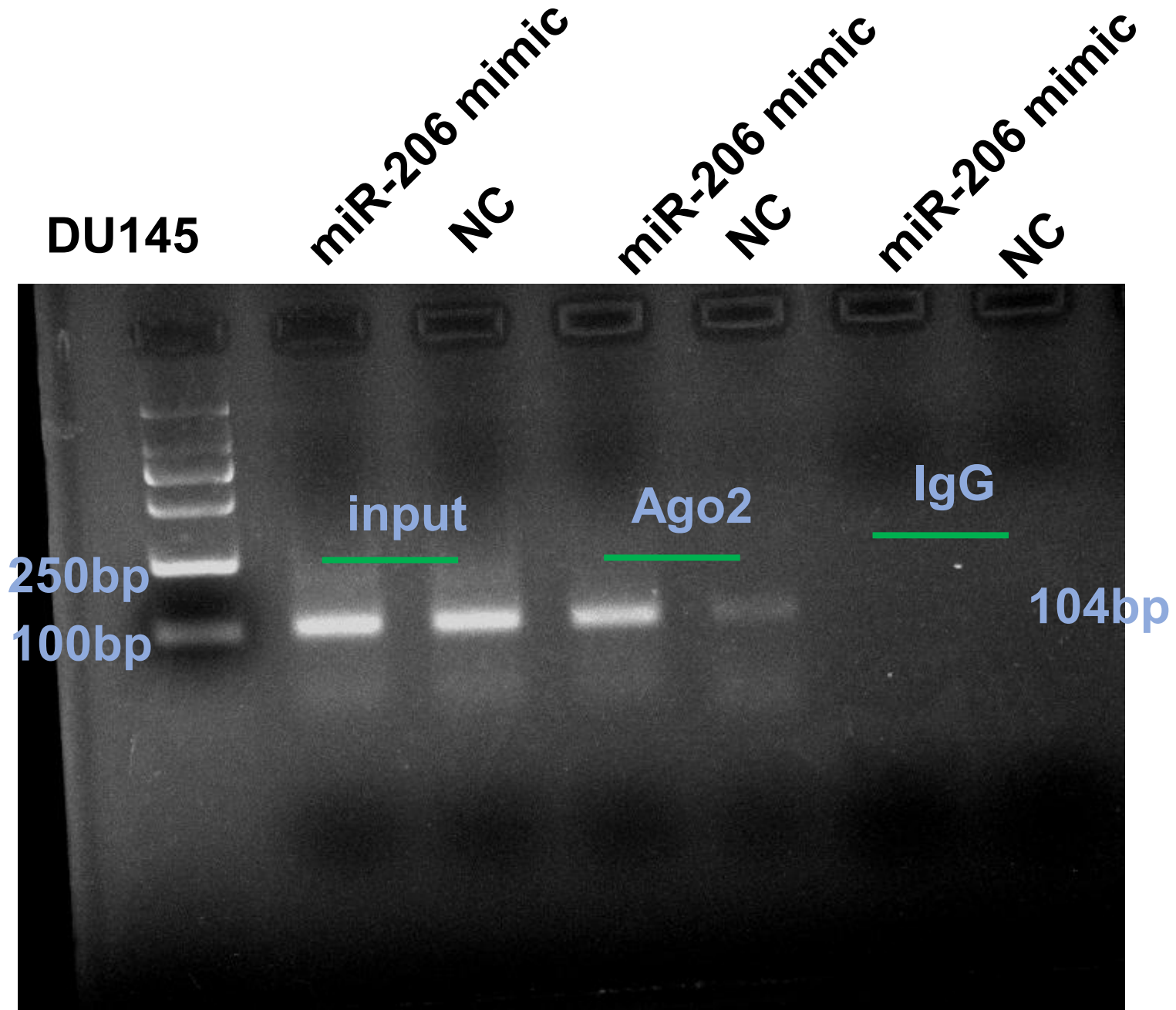

Figure 4B

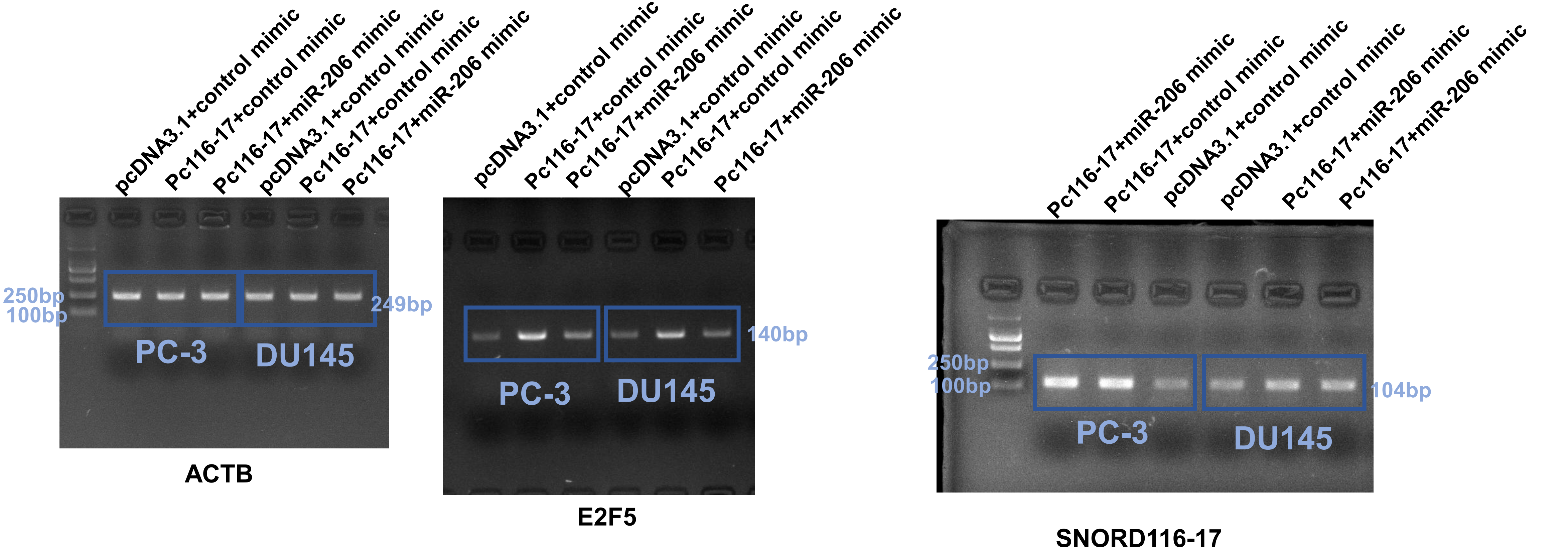

pcDNA3.1+control mimic
Pc116-17+control mimic
Pc116-17+miR-206 mimic
pcDNA3.1+control mimic
Pc116-17+control mimic
Pc116-17+miR-206 mimic
250bp
100bp
249bp
PC-3
DU145
ACTB
pcDNA3.1+control mimic
Pc116-17+control mimic
Pc116-17+miR-206 mimic
pcDNA3.1+control mimic
Pc116-17+control mimic
Pc116-17+miR-206 mimic
140bp
PC-3
DU145
E2F5
Pc116-17+miR-206 mimic
Pc116-17+control mimic
pcDNA3.1+control mimic
pcDNA3.1+control mimic
Pc116-17+miR-206 mimic
Pc116-17+miR-206 mimic
250bp
100bp
104bp
PC-3
DU145
SNORD116-17

RNA dot blot assay

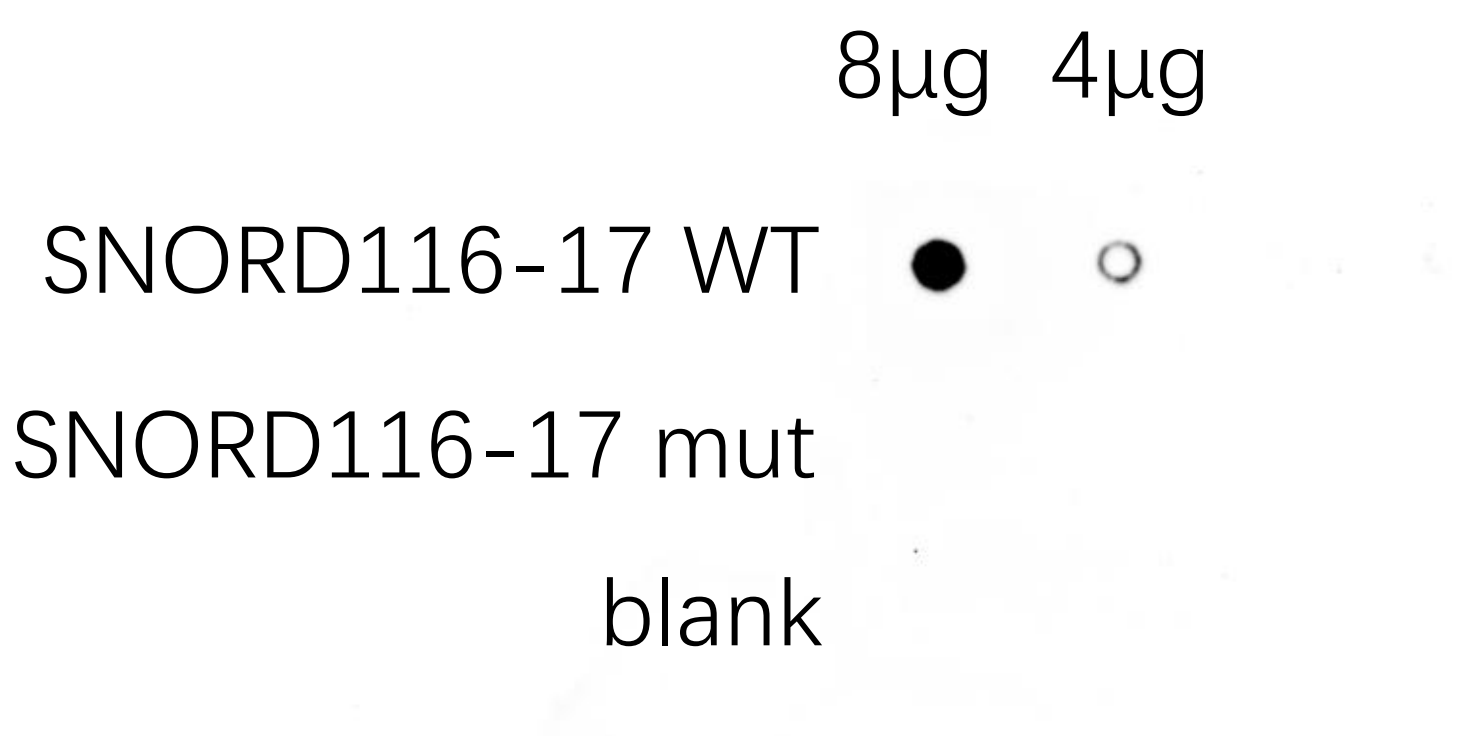

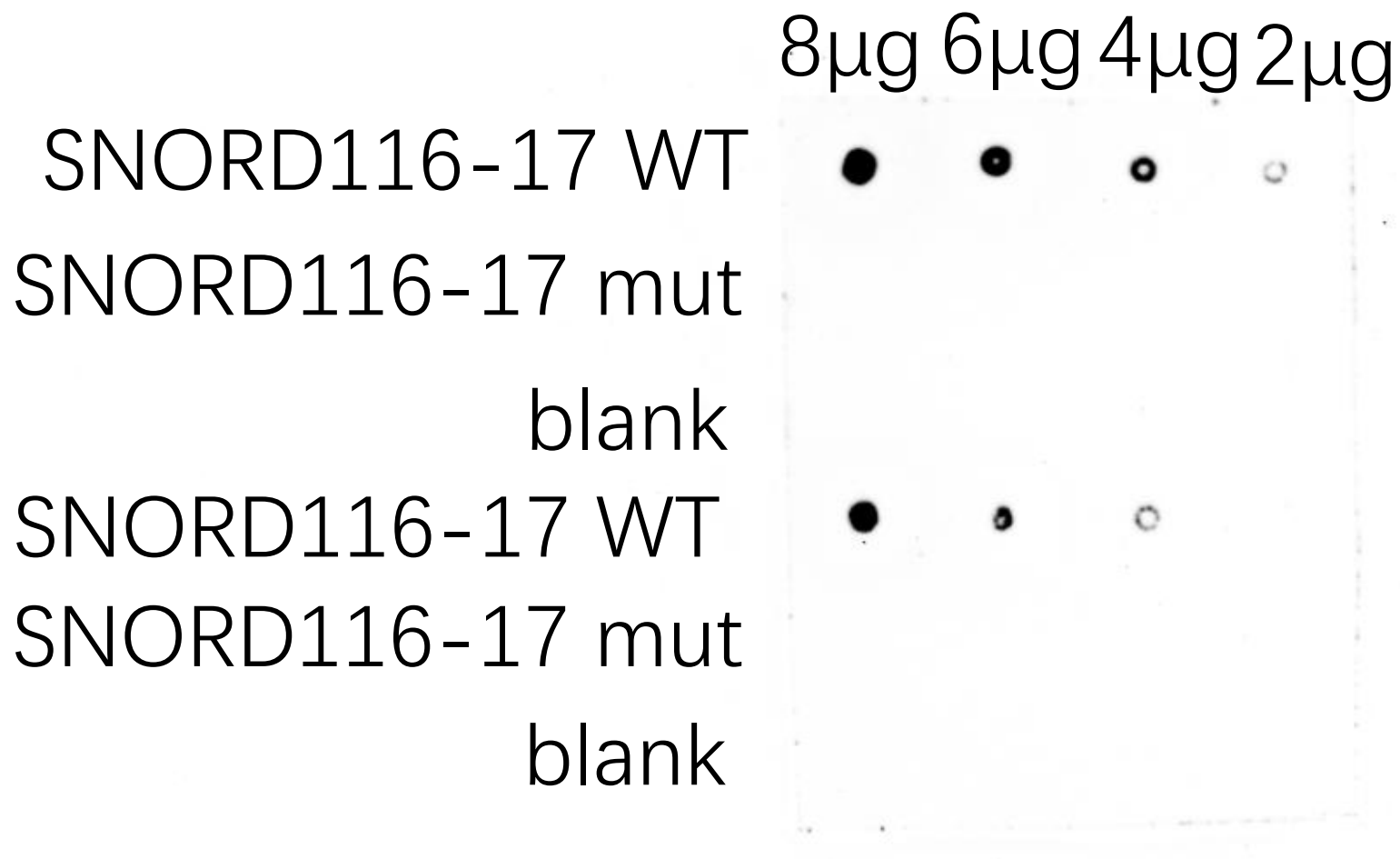

WB

FIG 3F

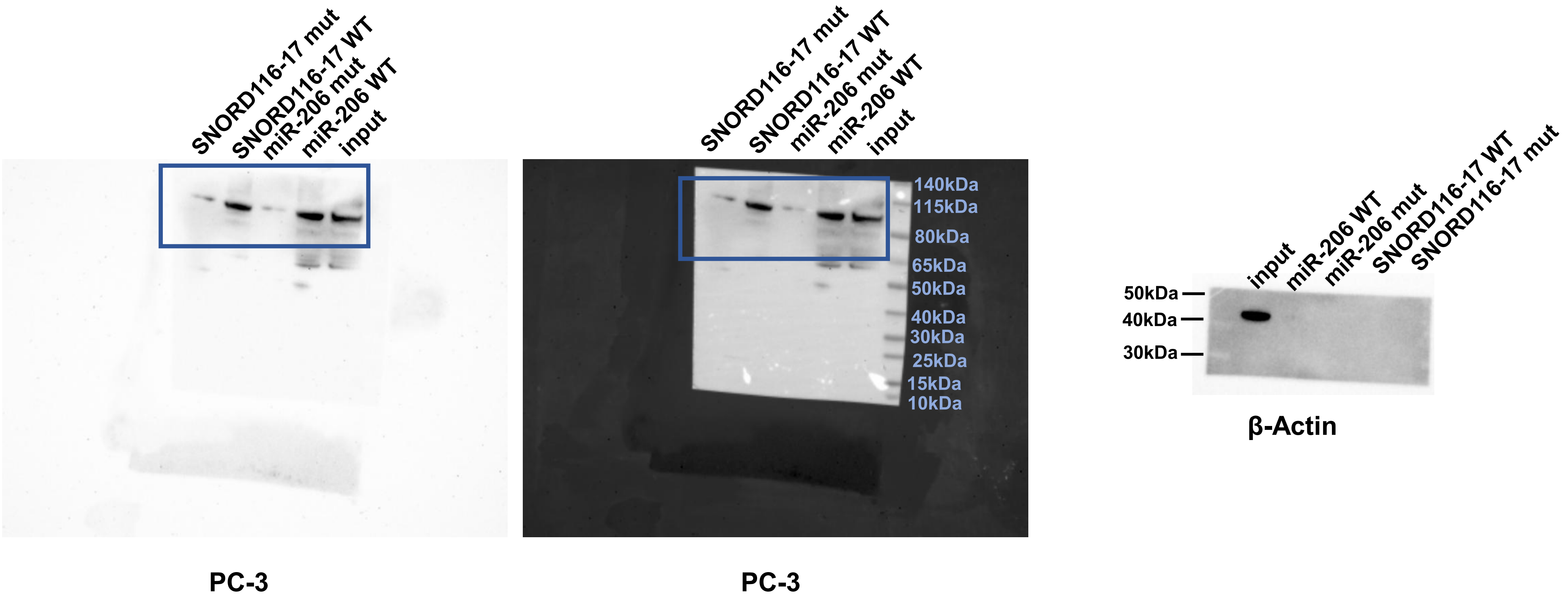

E2F5 and β-Actin were transferred onto separate membranes.

WB

FIG 3F

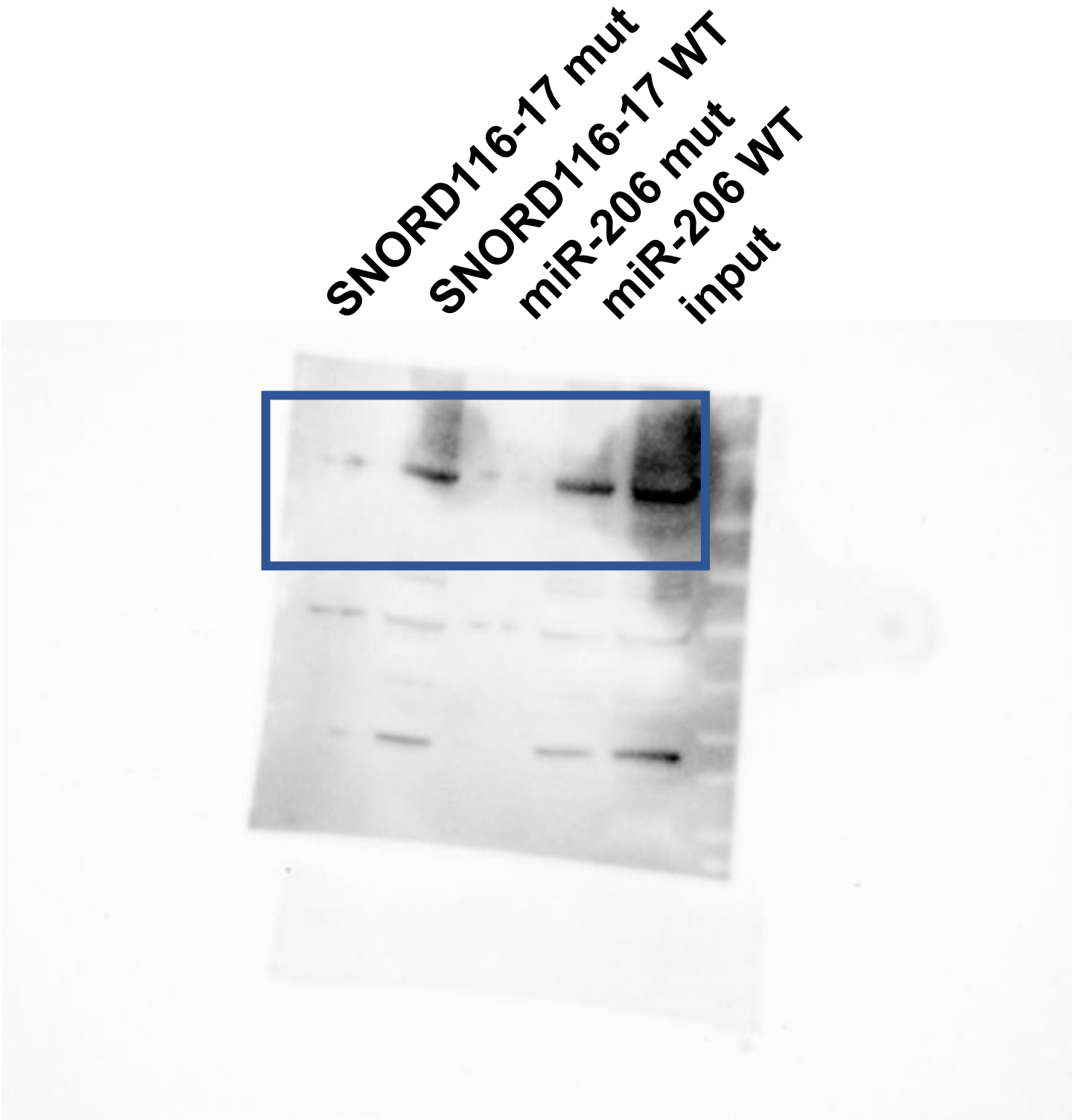

DU145

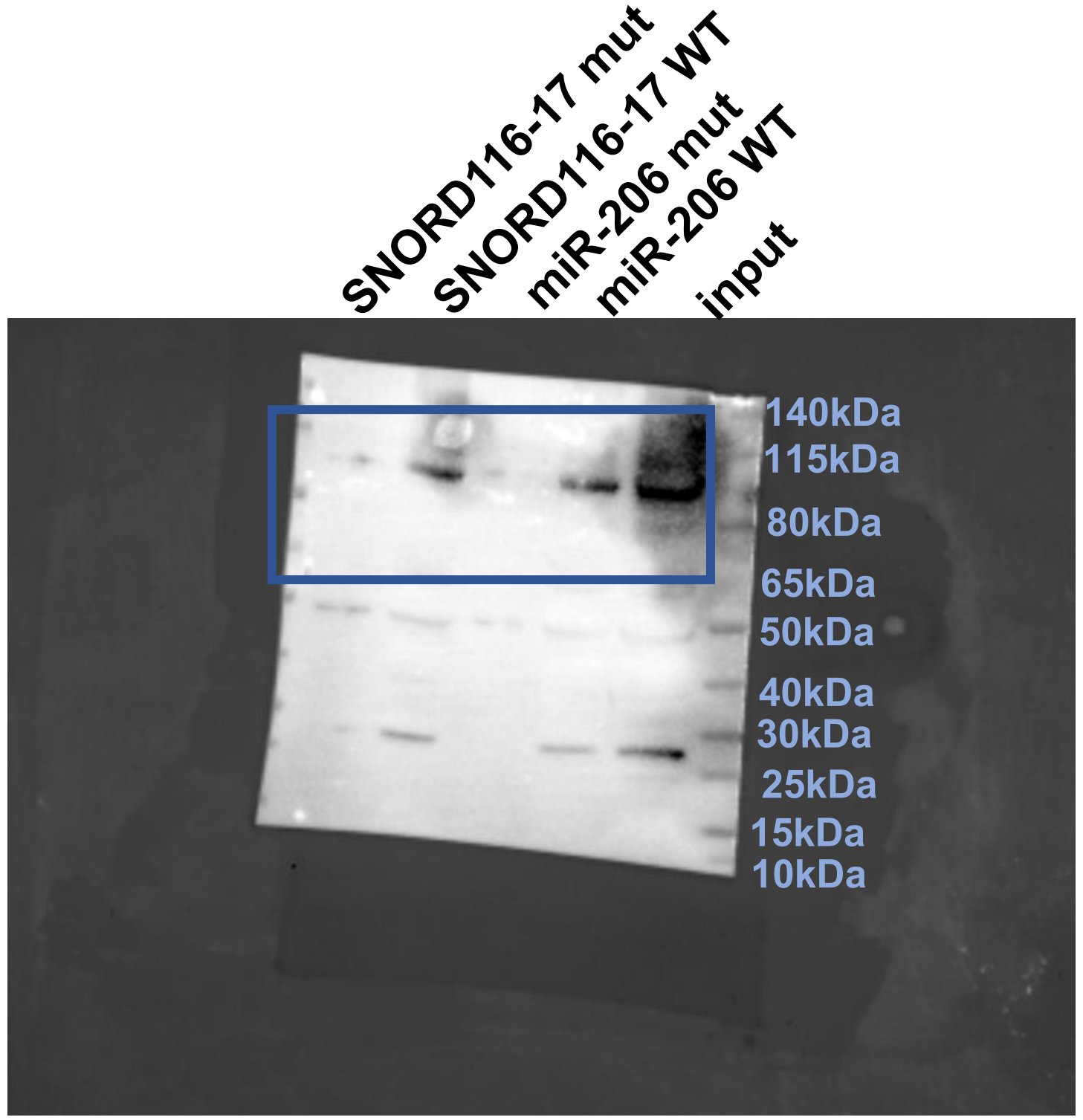

DU145

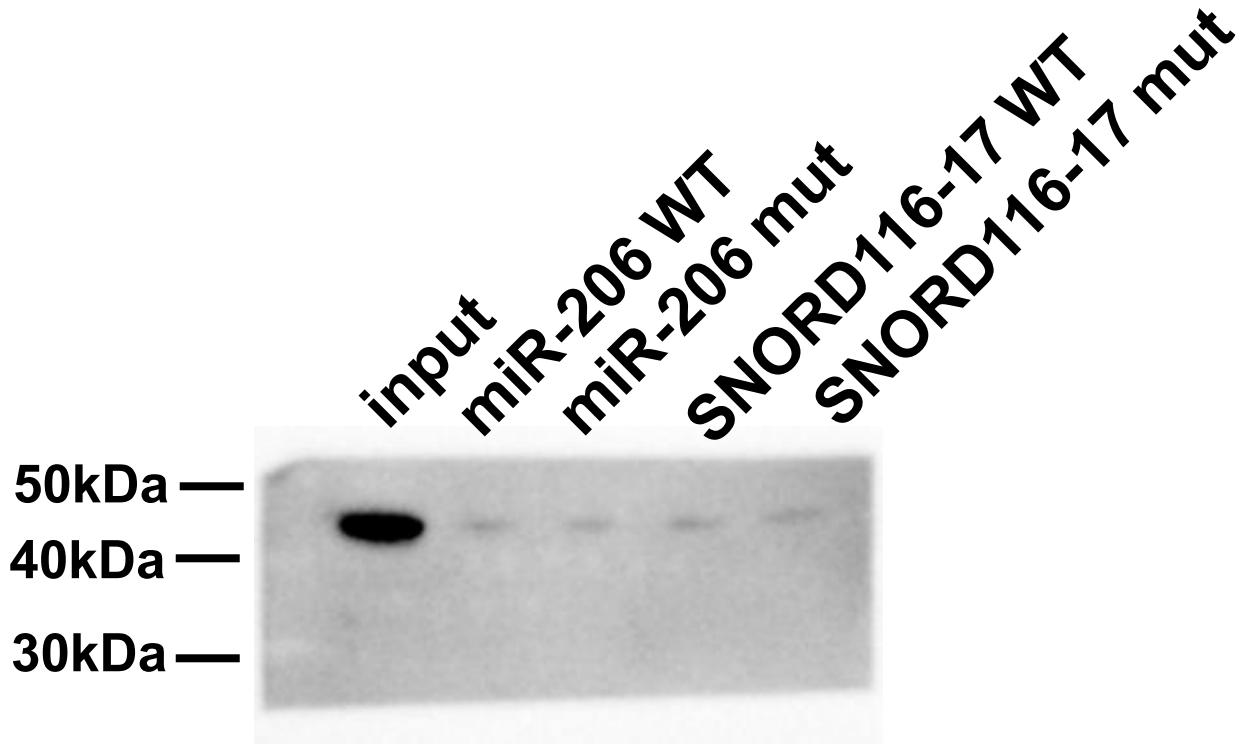

β-Actin

E2F5 and β-Actin were transferred onto separate membranes.

FIG 3K

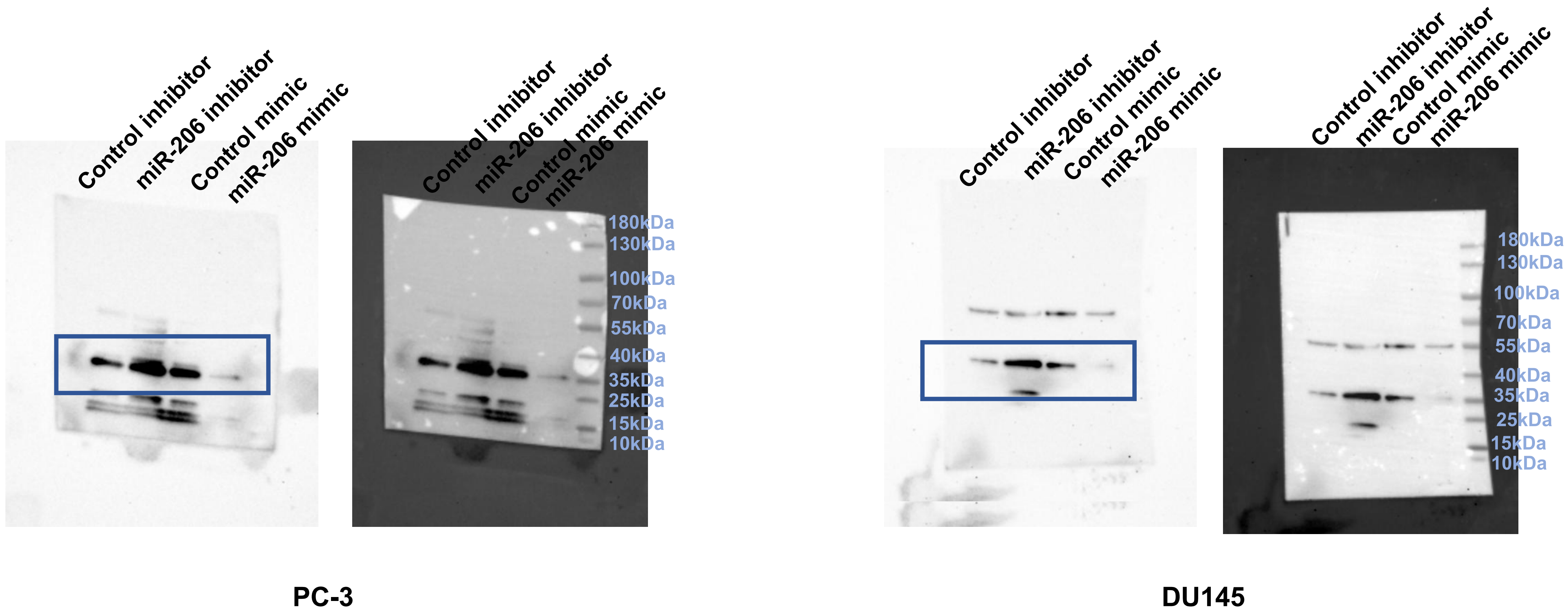
Control inhibitor
miR-206 inhibitor
Control mimic
miR-206 mimic
Control inhibitor
miR-206 inhibitor
Control mimic
miR-206 mimic
180kDa
130kDa
100kDa
70kDa
55kDa
40kDa
35kDa
25kDa
15kDa
10kDa
Control inhibitor
miR-206 inhibitor
Control mimic
miR-206 mimic
Control inhibitor
miR-206 inhibitor
Control mimic
miR-206 mimic
180kDa
130kDa
100kDa
70kDa
55kDa
40kDa
35kDa
25kDa
15kDa
10kDa
PC-3
DU145

FIG 3K

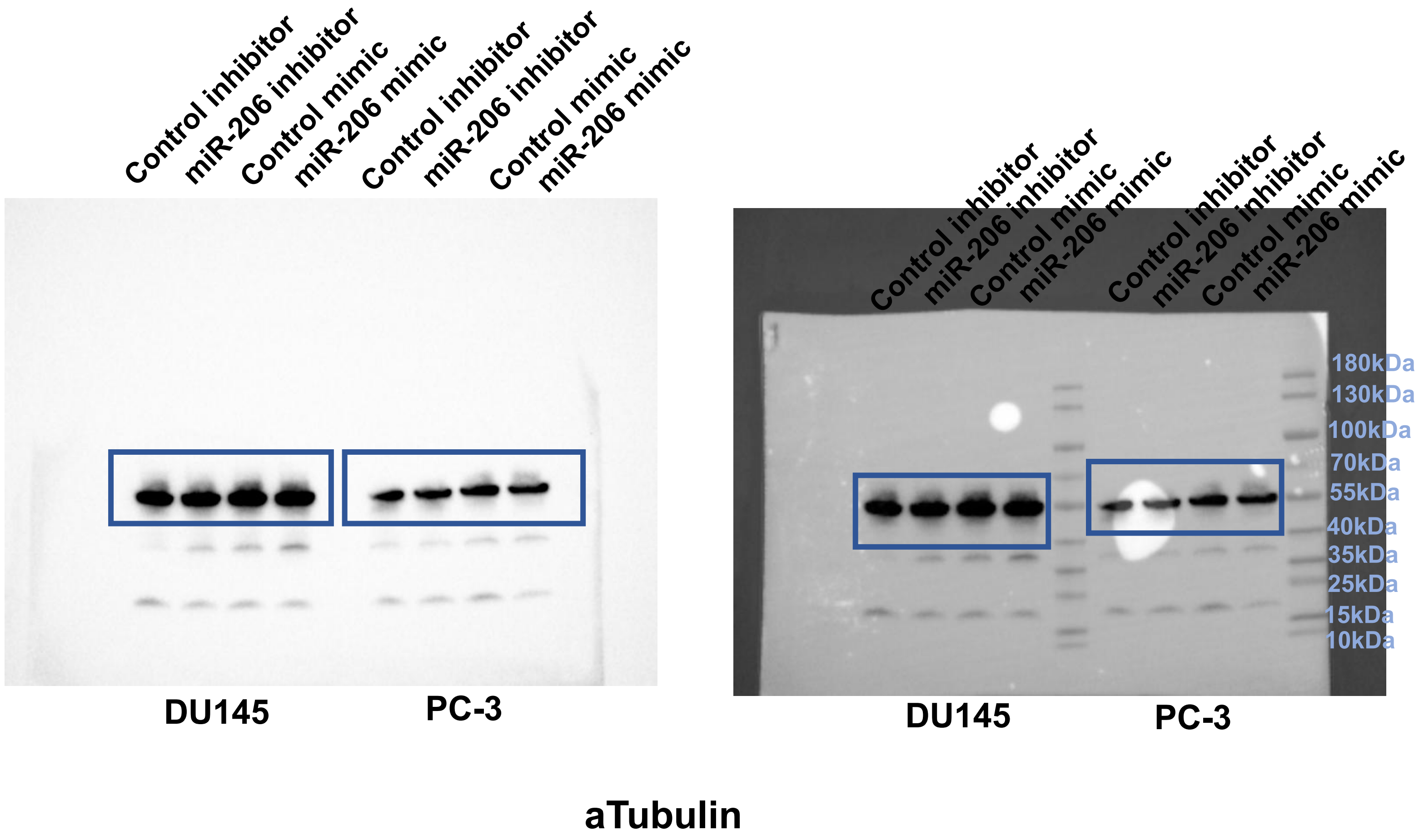

FIG 3K. E2F5 and α-tubulin were transferred onto separate membranes.

Figure 4C

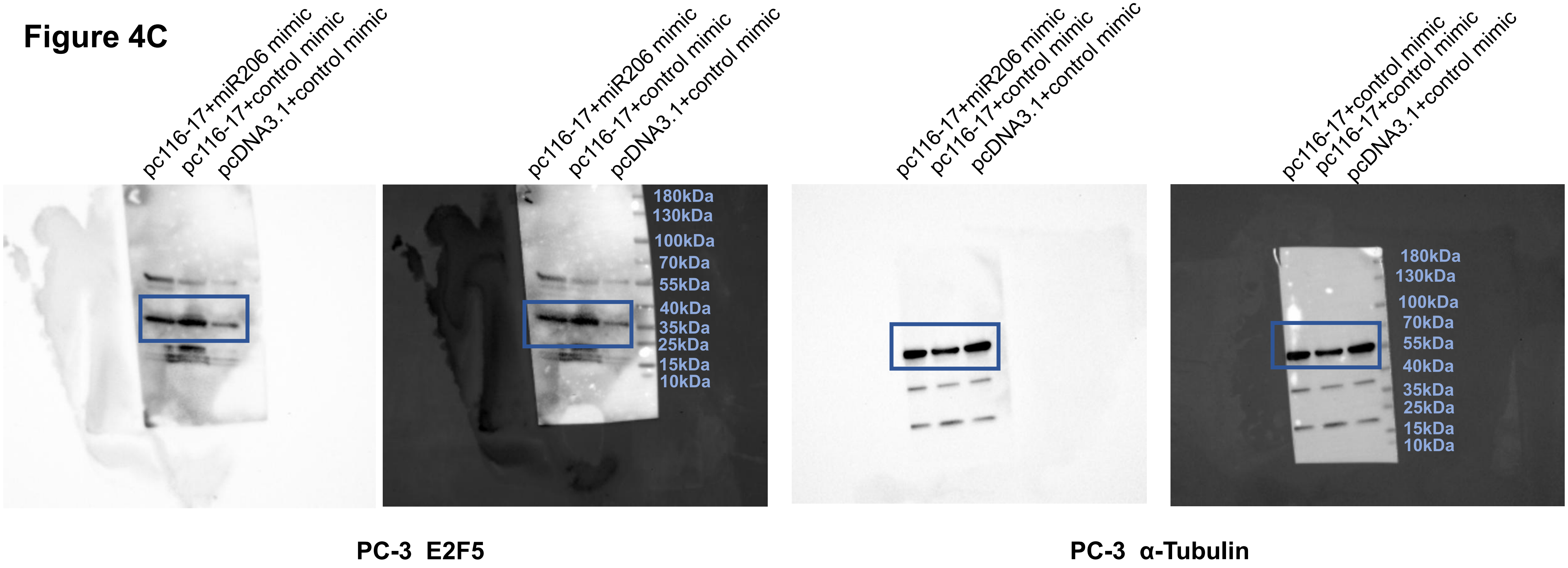

E2F5 and α-tubulin were transferred onto separate membranes.

Figure 4C

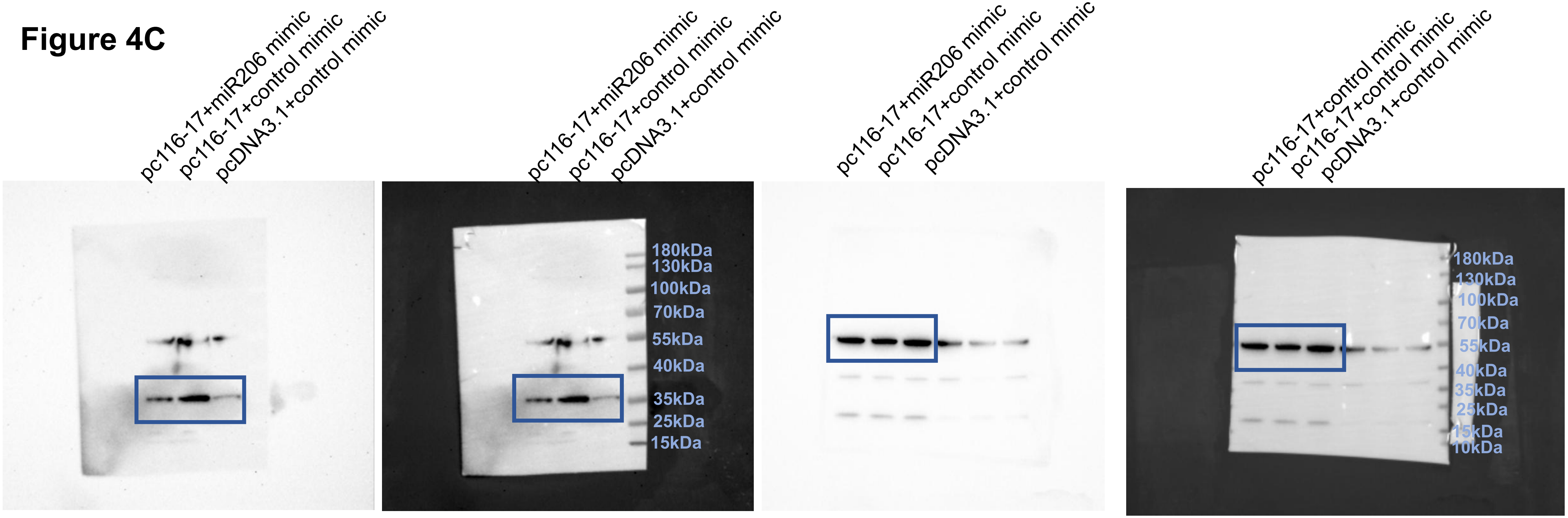

DU145 E2F5

DU145 a-Tubulin

E2F5 and α-tubulin were transferred onto separate membranes.

Figure 5C

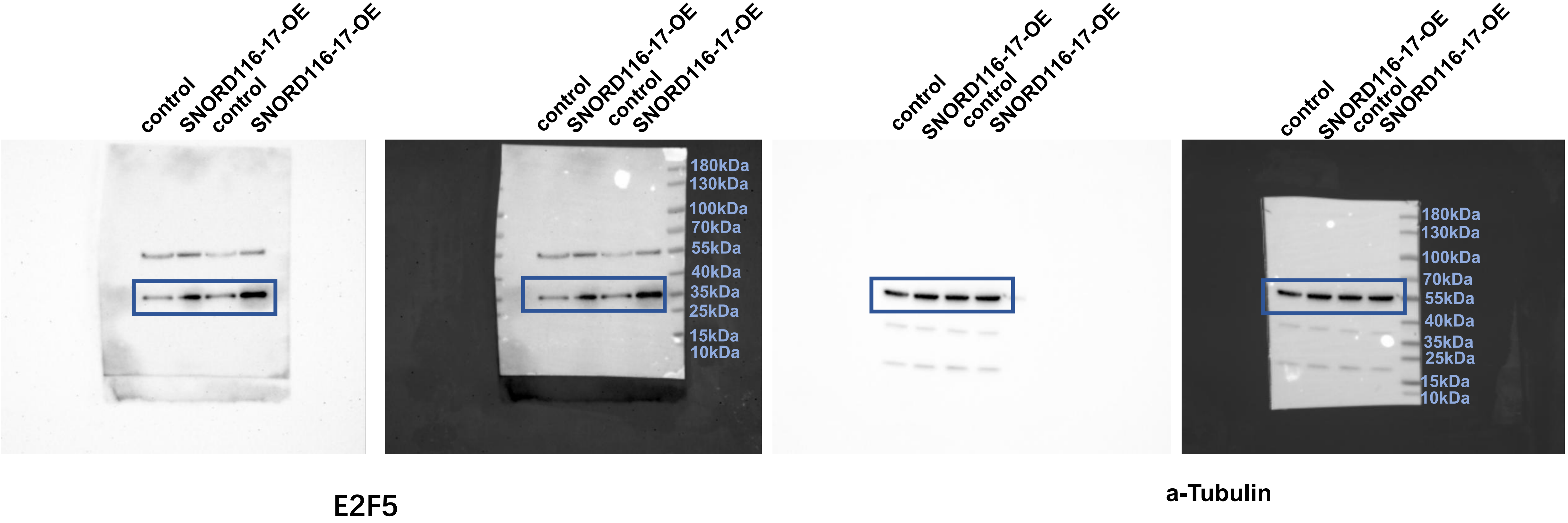

E2F5 and α-tubulin were transferred onto separate membranes.
